# Rapid, Matrix-Dependent Changes in Polyphenols and Antioxidant Capacity of Methanol Plant Extracts During Short-Term Storage: Implications for Analytical Timing

**DOI:** 10.3390/ijms27093723

**Published:** 2026-04-22

**Authors:** Attila Kiss, Tarek Alshaal

**Affiliations:** 1 Department of Food Biotechnology, Albert Kazmer Mosonmagyarovar Faculty, Széchenyi István University, Egyetem Sqr. 1, 9026 Gyor, Hungary; kiss.attila.peter@sze.hu; 2 Institute of Applied Plant Biology, Faculty of Agricultural and Food Sciences and Environmental Management, University of Debrecen, Boszormenyi Str. 138, 4032 Debrecen, Hungary

**Keywords:** antioxidant stability, pigment-rich vegetables, plant secondary metabolites, analytical protocol optimisation, oxidative degradation

## Abstract

Throughout this study, the short-term stability of methanol extracts was evaluated in cases of 15 distinctive, antioxidant-rich plant materials over 3, 7, and 14 days under refrigeration (4 °C), dark room-temperature, and light-exposed room-temperature conditions. A great variability in the matrix-dependent stability of the antioxidants, as well as the pronounced impact of the implied storage conditions on their plausible degradation, was revealed and featured. Initial total polyphenol content (TPC) ranged from 50.50 ± 0.44 mg gallic acid (GAE)/g DW (rosemary) to only 0.02 ± 0.006 mg GAE/g DW (amaranth). After 14 days, pigment-rich vegetable extracts (basil, beetroot powder, spinach powder, dried onion, tomato powder, and yarrow tail) lost 86.2–89.2% of TPC and 80–99% of DPPH (2,2-diphenyl-1-picrylhydrazyl) activity across all conditions, even under refrigeration. In contrast, for Lamiaceae species, markedly higher levels of the referred parameters were to be observed after 14-day-long storage. Decrease in TPC values was found to be 43.7% (rosemary), 50.6% (thyme), and 42.9% (oregano), respectively, while DPPH values were reduced by only 17–29%. Turmeric and walnut flour showed intermediate stability. Refrigeration consistently minimized the degradation of antioxidants (e.g., rosemary’s decrease in DPPH was only 20.3% at 4 °C vs. >70% under ambient conditions), while light exposure significantly accelerated losses of antioxidants in nearly all samples. Methanol extracts of many dietary plants, particularly pigment-rich ones, exhibit rapid and pronounced changes during short-term storage. Comparison with values obtained immediately after extraction shows that even brief storage can lead to substantial deviations. Although the current sampling intervals do not capture changes within the first hours, the results clearly indicate the need to minimize delays and standardize analytical timing to avoid underestimating phenolic content and antioxidant capacity. Moreover, these findings demonstrate that measured antioxidant properties are not solely inherent to the plant material but are strongly influenced by the extract matrix and methodological conditions. Consequently, antioxidant data should be regarded as matrix- and protocol-dependent, with important implications for their interpretation, comparability, and reproducibility across studies.

## 1. Introduction

People have long recognized the beneficial properties of plants and their derivatives [1]. Plant extracts and isolated compounds support health maintenance and amelioration, and remain integral to the pharmaceutical (e.g., cosmetics, vitamins) and food industries (e.g., spices, flavorings, additives). Among the myriad compounds in plants, many are biologically active and show promise for managing chronic diseases. These include pigments (e.g., carotenoids), fragrances and essential oils (e.g., Terpenoids), alkaloids, saponins, and numerous antioxidants [2,3,4]. Secondary metabolites with antioxidant activity encompass phenolic compounds, flavonoids, organic acids, pigments, and vitamins. Extensive evidence documents their health benefits [5,6,7,8]. Replacing synthetic antioxidants with plant-derived ones offers both technological and biological advantages [9,10,11].

The Lamiaceae family is rich in phenolics such as rosmarinic, salvianolic, and caffeic acids, and flavonoids including luteolin and apigenin; familiar species include rosemary, thyme, oregano, and basil [3,12,13]. Zingiberaceae species, notably turmeric, contain curcuminoids, turmerones, and gingerols [14,15]. Amaranthaceae members are valued for antioxidant pigments (betalains, betacyanins, chlorophyll, carotenoids), flavonoids, and phenolic acids [16,17,18]. Solanaceae plants, important in medicine and food, comprise alkaloids, tannins, flavonoids, terpenoids, saponins, and key compounds such as capsaicin, quercetin, and kaempferol [19,20,21]. Asteraceae species are abundant in terpenoids, flavonoids, phenolic acids, and carotenoids with high antioxidant activity [22,23,24]. Juglandaceae antioxidants include ellagic and gallic acids and flavonoids such as quercetin and kaempferol [25,26,27].

Fresh consumption of these antioxidant-rich plants is often impractical, necessitating paying extra attention to the proper storage conditions. Antioxidant activity declines over time under both natural and artificial conditions [28]. Short-term storage may lead to increased free-antioxidant content, whereas prolonged storage promotes decomposition of the relevant biomolecules [29]. Processed products sometimes differ from the fresh ones; free-antioxidant levels may rise during storage without a corresponding gain in the total antioxidant capacity [30]. Stability varies in accordance with the antioxidant classes and is influenced by temperature, light, oxygen, and pH.

Polyphenol degradation is accelerated at elevated temperatures via thermal oxidation, yielding less active compounds [31,32]. Processing above 80 °C generally reduces flavonoid stability, though some studies report on enhanced activity [33]. Anthocyanins are degraded at high temperatures, altering pigmentation [34]. Light, especially UV, induces isomerization and degradation of polyphenols [32]; colored light may elevate total phenolic content [35]. Flavonoid synthesis can be light-dependent, but prolonged exposure accelerates their breakdown [33,34].

Polyphenol instability is driven by oxidation; thus, oxygen-free or inert-gas environments are recommended [36]. Flavonoids show pronounced oxygen sensitivity [34], with pH-modulated oxidation rates [36]. Acidic conditions stabilize polyphenols, whereas alkaline media results in hastened decomposition and reduced antioxidant activity [32,36,37,38]. Discoloration accompanies these abovementioned changes across various compound classes [39].

Pre-storage treatments might influence the biological value (activity) of the investigated samples. Blanching may temporarily boost antioxidant capacity, but prolonged storage diminishes radical-scavenging ability [40,41]. The TPC of grape pomace extract proved to be stable across 20, 4, and −20 °C, while the DPPH capacity declined after 62 days [42]. Anthocyanins and phenolics were decomposed below 10 °C, but their contents were increased above this temperature [43]. In Lamiaceae, the amount of rosmarinic acid was decreased, while that of caffeic acid and total phenolics rose over 27 days, suggesting the formation of new antioxidants [44]. Exposure to blue light reduced the antioxidant content of strawberry due to anthocyanin instability [45]. Further research is needed to elucidate the complex features and intrinsic correlations affecting the stability of diverse plant–antioxidants in various matrices.

Beyond evaluating degradation patterns, this study is conceptually focused on disentangling the role of the extract matrix and methodological conditions in shaping the measured antioxidant properties. We hypothesize that the stability and apparent activity of antioxidants are emergent properties of complex chemical interactions within the extract, rather than fixed attributes of the original plant material. Therefore, variations in extract composition, solvent environment, storage, and analytical timing may substantially influence the obtained values, contributing to inconsistencies frequently reported in the literature.

The present study is engaged in surveying the alterations occurring in total polyphenol content (TPC) and total flavonoid content (TFC) in methanol extracts of 15 distinctive plant materials—rosemary, thyme, oregano, basil, turmeric powder, beetroot powder, amaranth, spinach powder, dried onion, tomato powder, chili pepper, red pepper spice, yarrow tail, walnut flour, and brewer’s yeast flakes—as a consequence of storage. Antioxidant capacity was assessed via DPPH, TEAC, and FRAP assays. The primary objective was to evaluate the effects of storage duration (3, 7, and 14 days) and conditions (refrigeration at 4 °C, dark at room temperature, and light-exposed shelf-storage at room temperature) on TPC, TFC, and antioxidant capacity. This is essential because analytical workflows are often based on temporary storage of the extracts, yet degradation risks compromise data validity and reproducibility. By establishing stability windows, the study determines whether immediate post-extraction analysis is mandatory, or if short-term storage is feasible without significant loss of the bioactive components to be analyzed, thereby optimizing laboratory protocols, reducing experimental bias, and ensuring reliable quantification of bioactive compounds in diverse, diet-relevant plant matrices. In this context, it is important to distinguish between truly immediate post-extraction conditions and short-term storage effects. Although baseline measurements were performed directly after extraction, the experimental design primarily captures changes occurring over storage periods of 3–14 days. Accordingly, the objective of the present study was not to define a precise stability threshold within the first hours after extraction, but rather to evaluate how rapidly deviations from initial values develop under commonly applied laboratory storage conditions. It should also be emphasized that the present investigation was conducted exclusively using extracts prepared with 100% methanol. While this solvent is widely employed in antioxidant screening workflows, it may influence oxidation kinetics and compound stability differently compared with aqueous or hydroalcoholic extraction systems. Therefore, the observed degradation patterns should be interpreted specifically in the context of methanol extracts and should not be directly generalized to plant extracts prepared using other solvent systems.

## 2. Results

### 2.1. Total Polyphenolic and Flavonoid Contents and Antioxidant Capacity in Herbs and Spices

Baseline values of TPC, TFC, and antioxidant capacity (DPPH, TEAC, FRAP) were determined immediately after extraction and are presented in Table 1. These values serve as reference points for evaluating subsequent changes during storage. Accordingly, all reported percentage losses reflect deviations from these initial measurements, allowing assessment of the extent to which storage alters the analytical outcomes relative to the original extract composition. Rosemary exhibited the highest TPC (50.5 ± 0.44 mg GAE/g DW), followed by thyme (41.9 ± 0.36), oregano (32.6 ± 1.96), walnut flour (29.9 ± 0.58), and yarrow tail (25.1 ± 0.10). Basil, turmeric powder, and tomato powder showed moderate TPC values of 24.2 ± 0.25, 19.3 ± 0.55, and 16.9 ± 0.86, respectively. Lower TPC levels were observed for chili pepper (8.3 ± 0.23), beetroot powder (7.8 ± 0.38), spinach powder (7.6 ± 0.60), red pepper spice (6.2 ± 0.61), dried onion (4.5 ± 0.44), and brewer’s yeast flakes (0.8 ± 0.06), while the lowest values were recorded for amaranth (0.02 ± 0.006) (Table S1; Supplementary Information). Turmeric powder displayed the highest TFC (148.9 ± 0.87 mg rutin/g DW), markedly surpassing values yielded for all the other species. Thyme followed with TFC values of 27.6 ± 0.43 (mg rutin/g DW), and then the following order was observed: rosemary (23.7 ± 0.36), red pepper spice (22.5 ± 0.27), yarrow tail (18.6 ± 0.11), and oregano (18.1 ± 0.25). The obtained TFC values for basil, tomato powder, spinach powder, chili pepper, and walnut flour ranged from 15.3 ± 0.32 to 5.6 ± 0.39. Beetroot powder, dried onion, and both amaranth and brewer’s yeast flakes exhibited the lowest TFC values, below 4.6 ± 0.06.

The antioxidant capacity via DPPH was found to be the highest in rosemary (487 ± 1.81 mg Trolox/g DW), followed closely by oregano (450 ± 0.50), thyme (449 ± 1.47), turmeric powder (447 ± 1.43), yarrow tail (438 ± 5.20), and walnut flour (424 ± 6.16). Basil showed a DPPH level of 340 ± 1.48 mg Trolox/g DW, while that of beetroot powder, red pepper spice, and chili pepper ranged from 294 ± 1.32 to 265 ± 3.01. DPPH values for amaranth were established to be 252 ± 2.25, followed by 112 ± 7.67 for tomato powder, 55 ± 5.15 for spinach powder, 41 ± 2.06 for dried onion, and 24 ± 4.77 for brewer’s yeast flakes.

In case of the TEAC assay (mg Trolox/g DW), rosemary again displayed the highest values with 10.2 ± 0.43, matched by turmeric powder and walnut flour with 9.9 ± 0.42 each, followed by thyme (9.7 ± 0.06), oregano (8.8 ± 0.51), and yarrow tail (7.7 ± 0.10). TEAC values gained for basil, red pepper spice, beetroot powder, and spinach powder ranged from 5.6 ± 0.21 to 4.4 ± 0.21. Dried onion and tomato powder both showed TEAC values of 2.9 ± 0.05–0.15, while chili pepper 2.1 ± 0.07, amaranth and brewer’s yeast flakes showed 1.2 ± 0.03–0.10.

FRAP values (mg ascorbic acid/g DW) proved to be the highest in rosemary (84.5 ± 3.15), followed by thyme and oregano (both 65.9 ± 0.84–1.35), basil (65.1 ± 2.46), turmeric powder (56.8 ± 1.62), walnut flour (55.6 ± 2.38), and yarrow tail (49.4 ± 1.51). For beetroot powder, the FRAP level of 40.9 ± 1.07 was recorded, while for spinach powder, 25.8 ± 0.99; for red pepper spice, 21.4 ± 0.73; for tomato powder, 18.9 ± 0.76; for chili pepper, 15.1 ± 0.52; and for amaranth, dried onion, and brewer’s yeast flakes, FRAP values ranged from 11.9 ± 0.46 to 11.0 ± 0.51.

### 2.2. Effect of Storage Period on Total Polyphenolic and Flavonoid Contents of Herbs and Spices

**Figure 1:**
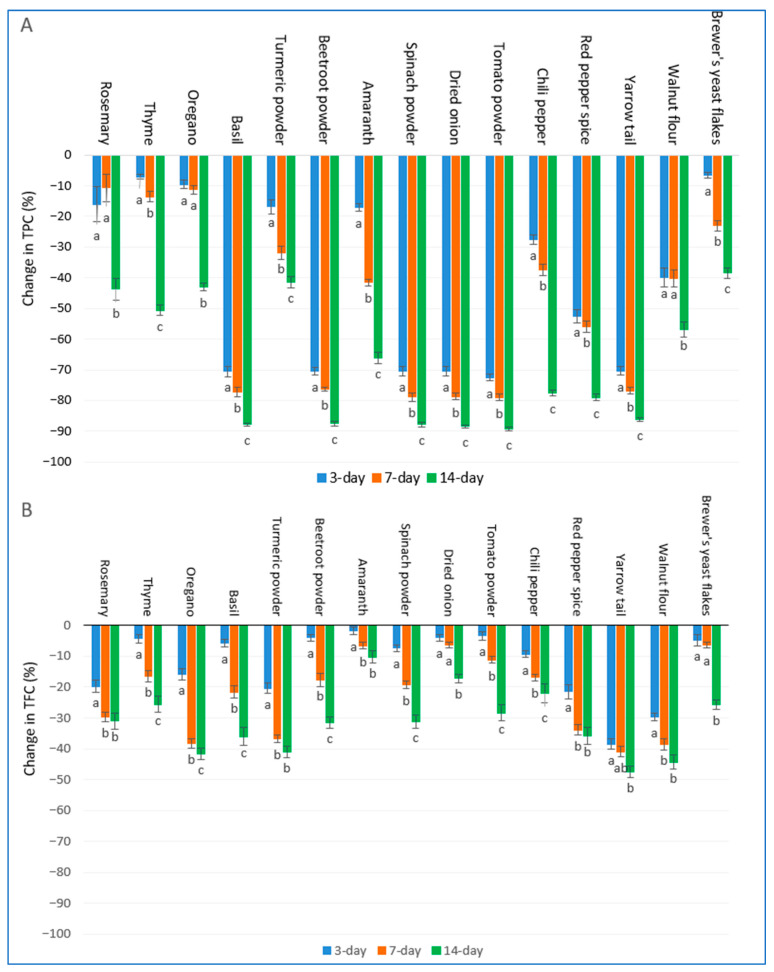
Changes in (**A**) total polyphenol content (TPC) and (**B**) total flavonoid content (TFC) in methanol extracts of 15 plant species under different storage periods. Bars sharing different letters within the same plant species indicate statistically significant differences at *p* ≥ 0.05 according to Tukey’s test. Values are presented as mean ± SD (n = 4).

All plant extracts exhibited progressive decline in TPC over the 14-day-long storage period, whose quantification was based on percentage losses calculated relative to fresh extracts (Figure 1A). After 3 days, basil, beetroot powder, spinach powder, dried onion, tomato powder, and yarrow tail showed the most pronounced reductions in terms of TPC levels, ranging from −70.3% to −72.6%. Red pepper spice (−52.4%), walnut flour (−39.8%), chili pepper (−27.6%), amaranth (−17.1%), turmeric powder (−16.9%), and rosemary (−16.0%) displayed moderate losses of polyphenols, while oregano (−9.6%), thyme (−7.1%), and brewer’s yeast flakes (−6.6%) proved to be the most stable in this respect. By day 7, degradation of polyphenols was intensified across most of the samples. Loss of polyphenols exceeded 76% in cases of basil (−77.2%), tomato powder (−79.0%), spinach powder (−78.9%), dried onion (−78.7%), beetroot powder (−76.3%), and yarrow tail (−76.9%). Red pepper spice (−56.0%), amaranth (−41.6%), walnut flour (−40.2%), and chili pepper (−37.4%) exhibited moderate loss of polyphenols with 37−56% reductions. Turmeric powder (−31.9%), brewer’s yeast flakes (−23.0%), thyme (−13.6%), oregano (−11.3%), and rosemary (−10.7%) showed relatively higher stability. After 14 days, losses were found to be the most severe in tomato powder (−89.2%), dried onion (−88.4%), beetroot powder (−87.6%), spinach powder (−87.8%), basil (−87.8%), and yarrow tail (−86.2%), all surpassing 86%. The decline of TPC exceeded 77% in cases of red pepper spice (−79.0%) and chili pepper (−77.7%), while amaranth (−66.1%) and walnut flour (−56.9%) showed 56–66% TPC reductions. The least degradation was experienced for thyme (−50.6%), rosemary (−43.7%), oregano (−42.9%), turmeric powder (−41.6%), and brewer’s yeast flakes (−38.4%), retaining over 49% of the initial TPC. It should be noted that “fresh extracts” refer to measurements conducted immediately after extraction (Table 1). However, as the first monitored time point in the present dataset occurs at day 3, the results describe early-stage storage effects rather than strictly immediate post-extraction kinetics within the first hours.

All plant extracts demonstrated time-dependent reductions in TFC during the 14-day-long storage period, with percentage changes expressed relative to initial values (Figure 1B). After 3 days, yarrow tail (−38.6%), walnut flour (−29.7%), turmeric powder (−20.3%), rosemary (−19.8%), and red pepper spice (−21.4%) exhibited the greatest losses of flavonoids. Moderate declines of TFC occurred in oregano (−15.8%), chili pepper (−9.3%), spinach powder (−7.3%), basil (−5.7%), brewer’s yeast flakes (−4.9%), thyme (−4.2%), beetroot powder and dried onion (both −4.0%), tomato powder (−3.3%), and amaranth (−1.8%). By day 7, the degradation was accelerated in most samples. Loss of flavonoids surpassed 29% in cases of yarrow tail (−41.0%), walnut flour (−38.7%), oregano (−38.3%), turmeric powder (−36.8%), red pepper spice (−33.9%), and rosemary (−29.7%). Basil (v21.6%), spinach powder (−19.3%), beetroot powder (−17.8%), chili pepper (−16.9%), and thyme (−16.6%) ranged from 16−22%, while tomato powder (−11.1%), amaranth (−6.6%), dried onion (−6.3%), and brewer’s yeast flakes (−6.2%) showed the highest stability of flavonoids, all detected below 12%. After 14 days, yarrow tail (−47.6%), walnut flour (−44.3%), oregano (−41.7%), turmeric powder (−41.1%), red pepper spice (−35.9%), and basil (−36.0%) recorded the most substantial reductions in flavonoids, exceeding 35%. The recorded decline in TFC values of rosemary (−31.0%), beetroot powder (−31.4%), spinach powder (−31.2%), tomato powder (−28.3%), thyme (−25.7%), and brewer’s yeast flakes (−25.7%) ranged from 25 to 31%, while that of chili pepper (−22.0%), dried onion (−17.2%), and amaranth (−10.2%) exhibited a lower extent of decline in terms of TFC.

### 2.3. Effect of Storage Period on Antioxidant Capacity of Herbs and Spices

Across all samples and assays, antioxidant activity consistently declined over time, with the most pronounced losses observed after 14 days of storage. The magnitude of decline varied considerably across plant species and the three distinct antioxidant assays applied.

**Figure 2:**
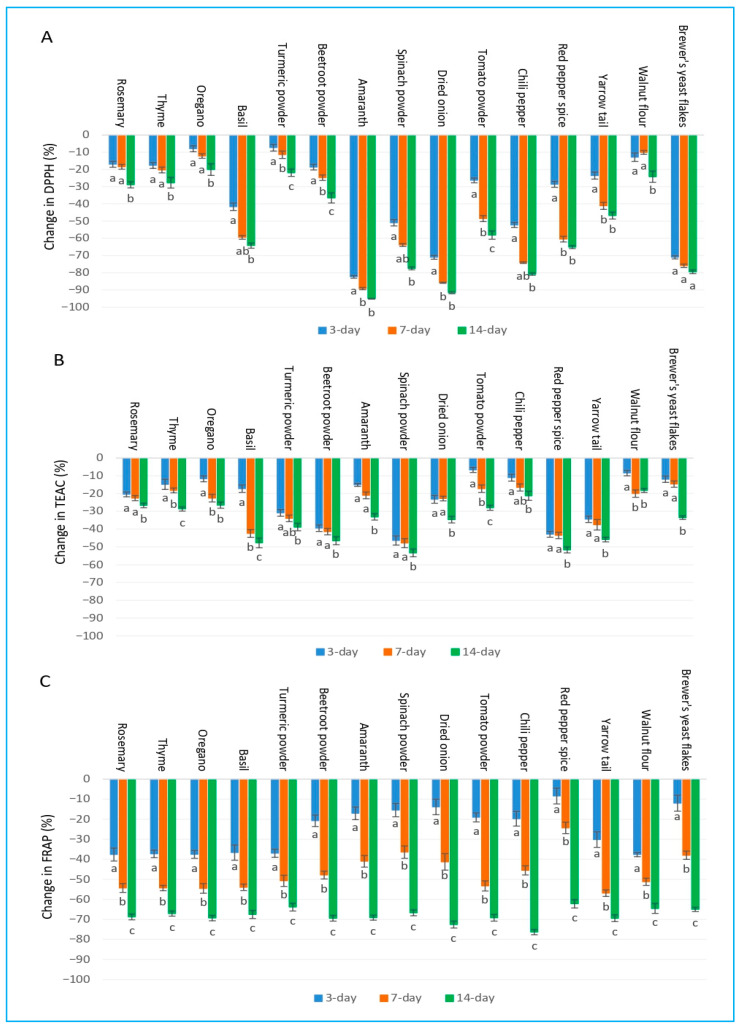
Changes in antioxidant capacity as measured by (**A**) DPPH, (**B**) TEAC, and (**C**) FRAP in methanol extracts of 15 plant species under different storage periods. Bars sharing different letters within the same plant species indicate statistically significant differences at *p* ≥ 0.05 according to Tukey’s test. Values are presented as mean ± SD (n = 4).

In terms of the implications of the DPPH assay, rosemary, thyme, and oregano showed the highest stability, with losses of 17–29% after 14 days, whereas amaranth, dried onion, brewer’s yeast flakes, and chili pepper exhibited the greatest antioxidant instability, exceeding 80% loss by day 14 (Figure 2A). Basil, spinach powder, red pepper spice, and tomato powder displayed intermediate to high degradation of the antioxidants (58–80% loss at 14 days).

In the case of the TEAC assay, degradation was generally less severe than that observed for DPPH in most samples. Rosemary, thyme, oregano, and walnut flour retained relatively high antioxidant activity (18–29% loss at 14 days), while beetroot powder, spinach powder, red pepper spice, and yarrow tail showed moderate to high losses of the antioxidants (45–54%). The TEAC values yielded for amaranth, dried onion, tomato powder, chili pepper, and brewer’s yeast flakes proved the least stable ones (Figure 2B).

By means of the FRAP assay the most dramatic reductions were revealed overall. Rosemary, thyme, and oregano again exhibited the most favorable behavior, though still losing 67–69% of the initial antioxidant activity by day 14. In the cases of the other samples, 62–76% losses were experienced, with beetroot powder and yarrow tail reaching approximately 69% degradation of the antioxidants, while several materials (basil, turmeric, amaranth, spinach, onion, tomato, chili, walnut, and brewer’s yeast) fell within a similar high-degradation range (Figure 2C).

Overall, rosemary, thyme, and oregano consistently demonstrated the greatest stability across all three applied antioxidant assays and the investigated storage time-periods, whereas amaranth, dried onion, brewer’s yeast flakes, chili pepper, and spinach powder were found consistently among the most labile materials after 14 days of storage.

### 2.4. Effect of Storage Conditions (Refrigerator, Dark, and Shelf) on Total Polyphenolic and Flavonoid Contents of Herbs and Spices

**Figure 3:**
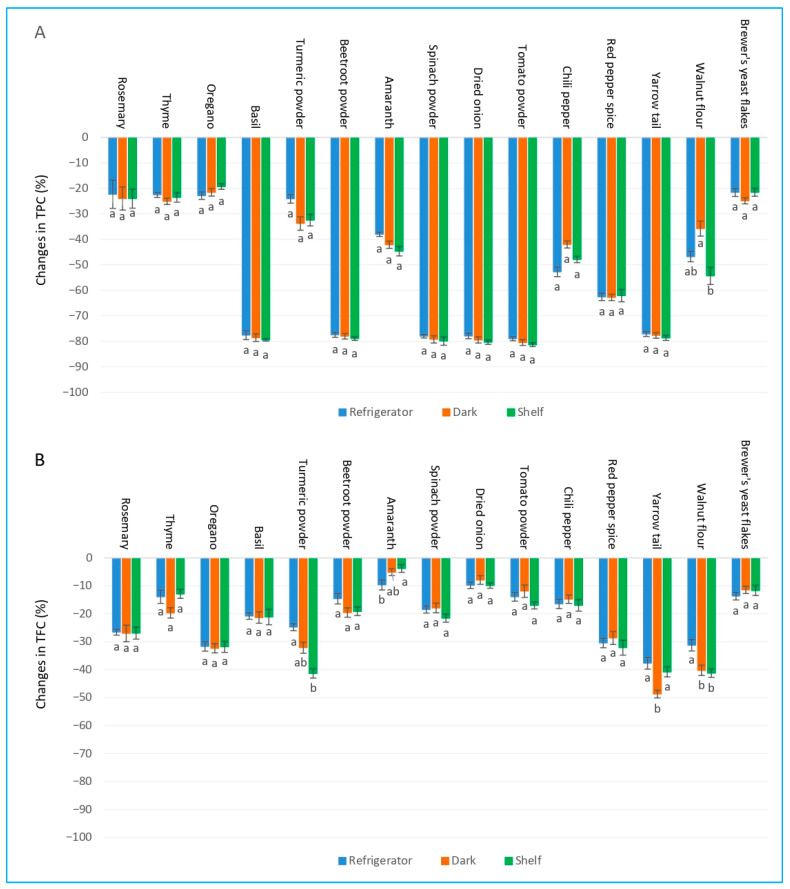
Changes in (**A**) total polyphenol content (TPC) and (**B**) total flavonoid content (TFC) in methanol extracts of 15 plant species under different storage conditions. Bars sharing different letters within the same plant species indicate statistically significant differences at *p* ≥ 0.05 according to Tukey’s test. Values are presented as mean ± SD (n = 4).

In terms of TPC, rosemary, thyme, oregano, turmeric powder, and brewer’s yeast flakes exhibited the highest stability, with losses ranging 19–34% across all the implied conditions (refrigerator, dark, and shelf) (Figure 3A). Thyme and oregano showed virtually no difference between the applied storage conditions (losses 19–25%). In contrast, basil, beetroot powder, spinach powder, dried onion, tomato powder, and yarrow tail suffered severe polyphenol degradation (77–81% loss), with minimal differences between refrigeration, dark, or light exposure. Moderate TPC losses (35–63%) occurred in amaranth, chili pepper, red pepper spice, and walnut flour, with walnut flour displaying the largest condition-dependent variation (35.8% dark vs. 54.3% light).

For TFC, the pattern differed markedly. Amaranth, dried onion, brewer’s yeast flakes, thyme, beetroot powder, chili pepper, and tomato powder showed excellent stability in terms of their flavonoid content, with losses generally below 20% under all conditions (Figure 3B). Rosemary and oregano retained their flavonoid contents to a relatively high degree (losses of 27–32%). Basil and spinach powder maintained moderate flavonoid stability (losses ~18–21%). The most pronounced flavonoid degradation occurred in cases of turmeric powder (especially under light: 41.3%), yarrow tail (up to 48.7% in dark), walnut flour (31–41%), and red pepper spice (28–32%).

Overall, phenolic compounds proved to be far more susceptible to the 14-day-long storage than flavonoids in most of the samples. Loss of TPC was largely independent of temperature and light conditions in highly labile materials (basil, beetroot, spinach, onion, tomato, yarrow), whereas TFC stability was generally high and often unaffected by light or temperature, except for turmeric, yarrow tail, and walnut flour, where light or room-temperature storage accelerated flavonoid degradation. Rosemary, thyme, oregano, and brewer’s yeast flakes demonstrated the best retention of both TPC and TFC across all tested conditions.

### 2.5. Effect of Storage Conditions (Refrigerator, Dark, and Shelf) on Antioxidant Capacity of Herbs and Spices

**Figure 4:**
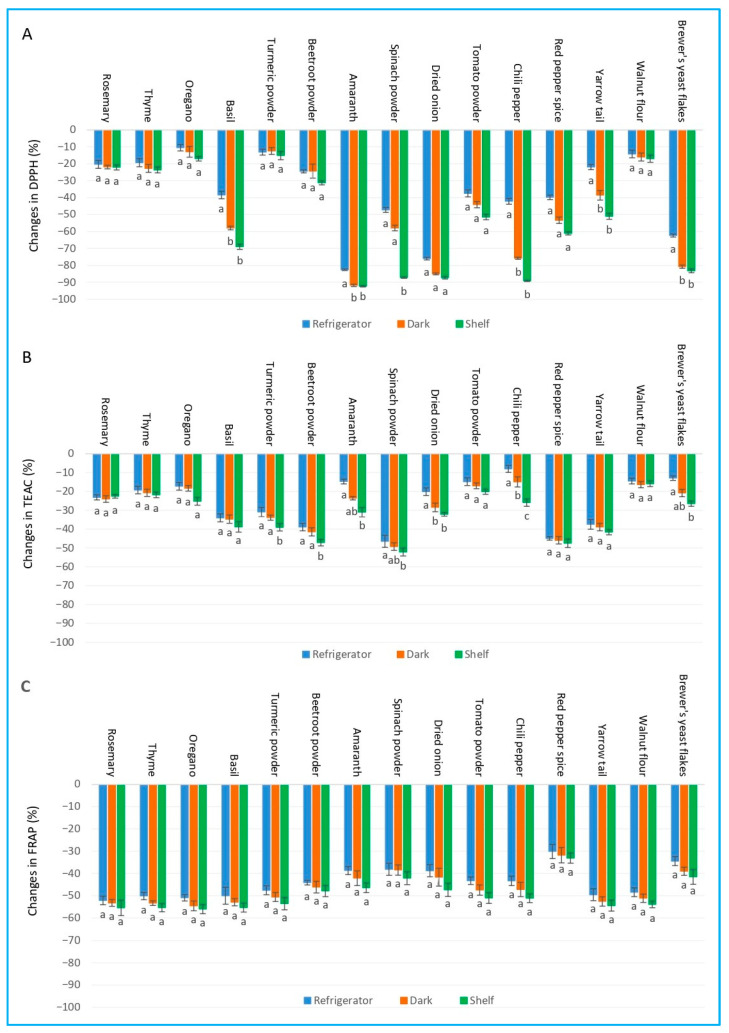
Changes in antioxidant capacity as measured by (**A**) DPPH, (**B**) TEAC, and (**C**) FRAP in methanol extracts of 15 plant species under different storage conditions. Bars sharing different letters within the same plant species indicate statistically significant differences at *p* ≥ 0.05 according to Tukey’s test. Values are presented as mean ± SD (n = 4).

Storage conditions (refrigerator, dark, and shelf) significantly impacted the antioxidant capacity of methanol extracts, as measured by the DPPH, TEAC, and FRAP assays. The decline of antioxidant activity followed a consistent pattern across most of the samples, with the most significant losses observed under light-exposed ambient conditions, followed by dark ambient conditions, and the least degradation under refrigeration (Figure 4).

Certain samples demonstrated high sensitivity to storage. Amaranth, spinach powder, and chili pepper exhibited the most substantial reductions in DPPH radical-scavenging activity, with losses reaching up to −92.3%, −87.2%, and −89.2%, respectively, when stored on a shelf. In contrast, extracts made from rosemary, thyme, and walnut flour were found to be more stable, particularly under refrigeration, showing DPPH declines as low as −20.3%, −19.3%, and −14.2%.

The rate of degradation varied significantly between the three antioxidant assays. For instance, amaranth extract showed a drastic decrease in terms of DPPH radical-scavenging activity (−82.6% to −92.3%), but a comparatively minor decline in TEAC activity (−14.7% to −31.0%). Similarly, basil exhibited a large decrease in DPPH and FRAP values, but a smaller change in TEAC antioxidant activity. Brewer’s yeast flakes also showed a high diminishment in DPPH radical-scavenging activity (−62.3% to −83.2%) but a much lower decrease in TEAC values (−12.9% to −26.4%). Conversely, red pepper spice showed the greatest relative reduction in TEAC values (−44.9% to −47.4%) compared to its DPPH and FRAP values. FRAP values generally showed a moderate and consistent decline across all the samples and conditions, with beetroot powder and red pepper spice exhibiting the smallest losses of antioxidants.

### 2.6. Effect of Storage Period and Conditions on Antioxidant Capacity of Herbs and Spices

After 3 days of storage, TPC declines ranged from minimal (−0.7% in brewer’s yeast flakes under refrigeration) to severe (−74.7% in tomato powder on the shelf). Materials richest in anthocyanins or chlorophyll-derived pigments (basil, beetroot, spinach, dried onion, tomato, yarrow tail) typically showed the largest initial TPC declines (68–75%), whereas spice herbs (rosemary, thyme, oregano) and turmeric lost only 3–27% of their polyphenol content. By day 14, reductions in TPC exceeded 80% in most pigment-rich materials (basil 87–88.7%, beetroot 87–88.7%, spinach 87.3–88%, dried onion 88–88.7%, tomato 88.7–89.7%, yarrow tail 86–86.7%), while rosemary, thyme, oregano, and turmeric retained relatively more phenolics (34–52% loss). Storage conditions had a significant and major effect on TPC for 11 of 15 species. It was observed that light-exposed shelf conditions generally accelerated polyphenol degradation greatly, compared to application of refrigeration or dark ambient storage. The TFC values showed even more pronounced decline over time. After 14 days, decomposition of polyphenols frequently exceeded 40–50% (e.g., oregano 40–43%, turmeric 34–53%, walnut 38–48%), and light exposure consistently amplified this phenomenon (significant Condition × Days interaction in 12 of 15 species).

Antioxidant capacity followed similar trends with marked assay- and species-specific differences. DPPH radical-scavenging activity displayed the most dramatic declines in several materials, reaching 95–99% reduction by day 14 in amaranth, spinach, dried onion, chili pepper, and brewer’s yeast flakes, especially under light exposure. In contrast, rosemary, thyme, oregano, and turmeric largely retained DPPH activity (final decreases of 58–72%). Light exposure strongly accelerated the diminishment of the DPPH values in nearly all cases (significant effect of the implied conditions in 13 of 15 species). TEAC values declined more gradually than those of DPPH in most of the species. The largest decreases occurred in pigment-rich materials (spinach, beetroot, shelf-stored chili and red pepper), reaching 50–57% reduction by day 14, while rosemary and thyme retained relatively higher TEAC values (25–29% reduction). FRAP (reducing power) showed the clearest time-dependent decline with relatively small influence of light in cases of many species. By day 14, the decrease in FRAP values ranged from 60 to 72% across almost all materials, with chili pepper, dried onion, and shelf-stored samples occasionally exceeding 80% reduction.

Overall, refrigeration at 4 °C consistently provided the best conditions for preservation of phenolic compounds and antioxidant capacity across the 14-day-long period, followed by dark ambient storage, while exposure to light at room temperature accelerated the degradation of both phenolics and flavonoids in nearly all tested plant extracts, leading to reduced antioxidant activities. Notably, the magnitude and direction of these changes cannot be explained solely by plant identity but rather reflect matrix-specific effects arising from the chemical composition of the extracts. The contrasting behavior of pigment-rich versus phenolic-rich samples suggests that interactions among co-extracted compounds, as well as their differential susceptibility to environmental factors, play a decisive role in determining both stability and measured antioxidant capacity.

### 2.7. Covariance Analysis of Interdependent Antioxidants

Significant variation among species dominated all measured parameters, with the strongest effects being observed for DPPH and TPC, as indicated by the highest covariance values for species (e.g., 4843.82 for DPPH; 3974.32 for TPC) (Table 2). Storage time exerted an even greater influence, particularly on FRAP (8069.84), TPC (6434.54), and TFC (3144.83), showing that antioxidant metrics changed markedly across the 3-, 7-, and 14-day-long intervals. Storage conditions had smaller but significant effects on all parameters, with the most pronounced influence on DPPH (2426.74) and TEAC (439.97), while interactions between days and conditions remained comparatively modest, yet still significant across all assays. The three-way interaction between species, days, and conditions was significant for every measured variable, confirming that degradation or stability patterns differed substantially among plant materials depending on storage duration and environment.

### 2.8. Multivariate Assessment of Antioxidant Stability via Correlation and PCA

**Figure 5:**
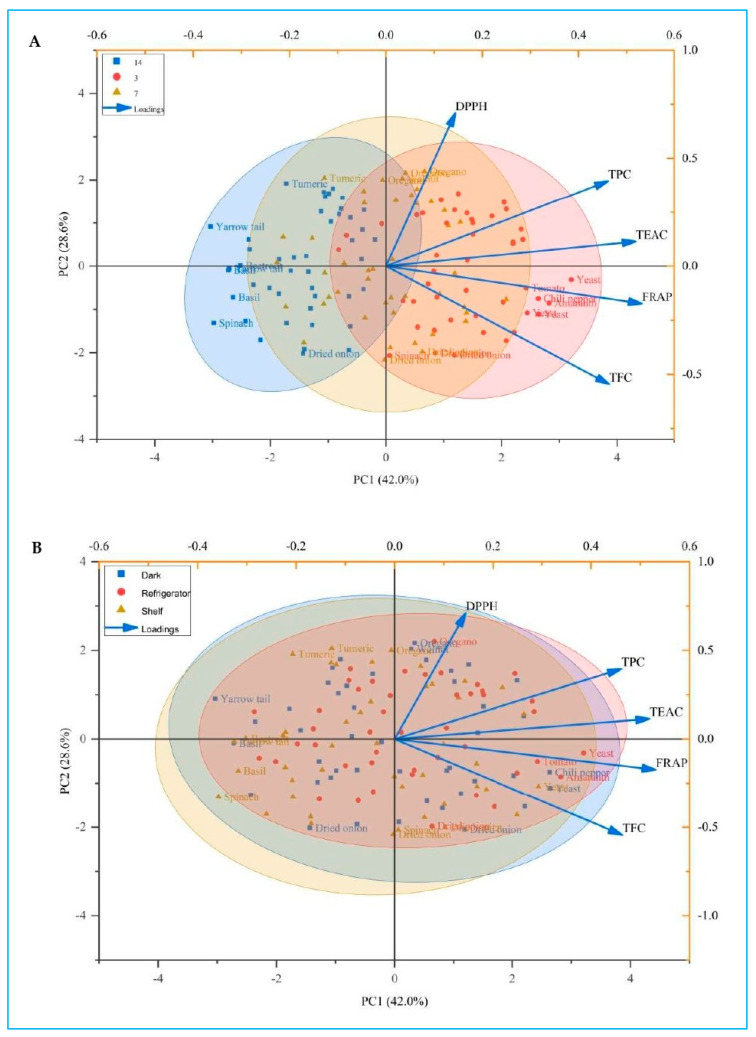
Principle component analysis of (**A**) storage period and (**B**) storage conditions on total polyphenols (TPC), total flavonoid content (TFC), and antioxidant capacity as measured by DPPH, TEAC, and FRAP in methanol extracts of 15 plant species—rosemary, thyme, oregano, basil, turmeric powder, beetroot powder, amaranth, spinach powder, dried onion, tomato powder, chili pepper, red pepper spice, yarrow tail, walnut flour, and brewer’s yeast flakes—after storage for 3, 7, or 14 days under different conditions: refrigeration (4 °C in a Whirlpool W7X 820 OX unit), dark ambient (22 ± 2 °C in a light-excluded cabinet), or light-exposed ambient (shelf; 22 ± 2 °C near a window) for different periods.

Correlation results indicate that storage duration showed strong negative relationships with nearly all antioxidant metrics, most notably FRAP (r = −0.889 **) and TFC (r = −0.537 **), demonstrating pronounced declines with prolonged storage (Figure 5). TPC displayed a moderate negative correlation with days (r = −0.406 **), while its relationships with antioxidant assays were positive, especially with TEAC (r = 0.555 **) and DPPH (r = 0.349 **). TFC correlated positively with FRAP (r = 0.564 **) and TEAC (r = 0.388 **), while showing a negative correlation with DPPH (r = −0.307 **). DPPH correlated negatively with both days and conditions, and positively with TPC (r = 0.349 **). TEAC correlated positively with all antioxidant metrics and TPC but negatively with storage period (r = −0.403 **). FRAP exhibited positive correlations with all compositional variables and antioxidant assays but declined with both storage duration and storage condition.

Scores for all samples cluster tightly around the origin on both PC1 (42.0%) and PC2 (28.6%), indicating that storage conditions and storage duration produce only modest shifts in the measured chemical attributes. Loadings show that TPC, TFC, TEAC, FRAP, and DPPH contribute positively to PC1, with TPC, TEAC, and DPPH exerting the strongest influence, while TFC and FRAP load moderately and are directed slightly downward on PC2.

Under different storage conditions, samples stored in the dark, under refrigeration, or on the shelf overlap extensively, reflecting minimal separation. Refrigerated samples distribute slightly farther along the positive direction of PC1, aligning more closely with higher TPC, TEAC, and DPPH. Shelf-stored samples display a broader spread, while dark-stored samples are positioned more toward the negative-PC1 side, suggesting relatively lower antioxidant metrics. Individual plant species show natural clustering consistent with their inherent composition—oregano, turmeric, and walnut flour gravitate toward the positive-PC1 region associated with stronger antioxidant activity, while spinach powder, basil, and dried onion group toward the negative region.

Across storage duration, samples taken at 3, 7, and 14 days again form overlapping clusters, indicating stability of TPC, TFC, and antioxidant capacity over time. Day-3 samples tend to shift slightly toward the positive PC1 axis, mapping closer to higher antioxidant loadings, whereas day-14 samples occupy more negative PC1 values, reflecting a modest decline in these traits. Day-7 samples fall between the two clusters, consistent with intermediate values. As in the storage condition plot, oregano, turmeric, and walnut flour align with stronger antioxidant vectors, whereas spinach, basil, and dried onion remain positioned in regions of lower values.

## 3. Discussion

### 3.1. Rapid Decline in Antioxidant Capacity

The present study reveals that methanol extracts made from all the investigated 15 antioxidant-rich plant materials undergo rapid and substantial degradation of their phenolic compounds, leading to decreased antioxidant capacity during short-term storage of 3–14 days, regardless of refrigeration or darkness (Figures S1–S15; Supplementary Information). The magnitude of the decrease is strongly species-dependent. By day 14, reductions often exceed 85–90% in TPC and DPPH activity. In contrast, culinary herbs from the Lamiaceae family (rosemary, thyme, oregano) and turmeric retain a greater proportion of their antioxidant capacity. Over the same period, losses are limited to 34–52% for TPC and 58–72% for DPPH activity. Refrigeration at 4 °C consistently provides the strongest protection, followed by dark ambient storage, while exposure to normal laboratory light at room temperature markedly accelerates degradation of the antioxidants in nearly every extract. These findings have direct practical consequences for analytical laboratories handling plant extracts: for most of the materials, especially pigment-rich ones, immediate analysis after extraction is essential to avoid severe underestimation of the actual bioactive content.

Initial (day 0) TPC (mg GAE/g DW) values align closely with previously published data for methanol extracts. Rosemary displayed the highest TPC (50.5), followed by thyme (41.9) and oregano (32.6), confirming the exceptional richness of Lamiaceae species in rosmarinic acid, caffeic acid derivatives, and stable phenolic diterpenes such as carnosic acid and carnosol [12,13,46]. Turmeric and walnut flour also exhibited high phenolic content, in agreement with reports on curcuminoids and hydrolysable tannins [14,27]. In contrast, pigment-rich materials such as beetroot, spinach, tomato, and dried onion showed surprisingly low initial TPC values despite intense coloration, because many of their dominant pigments (betalains, chlorophyll derivatives, lycopene) react poorly with the Folin–Ciocalteu reagent, which primarily detects phenolic hydroxyl groups rather than all the reductants [47,48,49].

The most remarkable observation is the extraordinarily rapid decline in the TPC values in pigment-rich extracts, reaching 68–75% decrease already after 3 days and exceeding 86% after 14 days, with degradation of antioxidants being largely independent of temperature or light protection in these materials. This rapid decomposition in methanol solution far exceeds rates typically reported for solid dried herbs or aqueous infusions. In solution, residual dissolved oxygen and trace transition metals leached from the plant material trigger rapid non-enzymatic oxidation of catechol- and pyrogallol-containing phenolics (chlorogenic acid, quercetin glycosides, protocatechuic acid), transforming them into reactive o-quinones that polymerise into insoluble brown melanoid pigments, progressively removing soluble Folin-reactive compounds [31,32]. Disruption of natural co-pigmentation complexes upon extraction further exposes previously protected phenolics to oxidation, while the mildly acidic environment of many extracts (pH 4.0–5.5) promotes decarboxylation and esterification reactions that reduce the reactivity of phenolics [34,36,37]. In contrast, rosemary, thyme, and oregano exhibited markedly slower degradation of their antioxidants, because their dominant phenolics (carnosic acid, carnosol, rosmarinic acid) possess intrinsic antioxidant mechanisms: carnosic acid, for example, is sacrificially oxidized while regenerating itself through intramolecular cyclisation, thereby shielding neighboring molecules [46,50]. Turmeric’s relative stability similarly reflects the robustness of curcuminoids in the dark. Earlier studies on intact, dried Lamiaceae herbs or their infusions occasionally reported stable or even increasing phenolic content over weeks, due to continued hydrolysis of the bound forms [44], underscoring that solvent extraction removes the protective plant matrix and dramatically accelerates oxidative processes.

A slight increase in TPC between day 3 and day 7 was observed for rosemary extracts. This effect most likely reflects methodological and matrix-related factors rather than a real increase in phenolic content. In methanolic plant extracts, short-term increases in Folin–Ciocalteu-reactive compounds may occur due to delayed release of matrix-bound phenolics, improved solubilization of extractable components during storage, or formation of secondary oxidation products that retain reducing capacity and therefore contribute to the measured signal. Because the Folin–Ciocalteu assay determines total reducing capacity rather than exclusively native phenolic structures, such temporary increases can occur without contradicting the overall degradation trend observed during prolonged storage.

The TFC proved to be substantially more stable than TPC across most species, with many extracts preserving 70–90% of the initial TFC after 14 days, and significantly accelerated light-induced decomposition of the antioxidants observed only in turmeric, yarrow tail, walnut flour, and red pepper spice. This resilience arises because the dominant flavonoids in the more stable materials (thyme, oregano, rosemary, dried onion, brewer’s yeast) are flavones and flavanones lacking the catechol B-ring required for rapid auto-oxidation, whereas the light-sensitive extracts contain higher proportions of quercetin, kaempferol, and curcuminoid compounds that readily undergo photoisomerization or singlet-oxygen attack [51,52].

Antioxidant capacity followed distinct patterns depending on the assay, reflecting the existence of different viable degradation pathways. DPPH radical-scavenging activity displayed the most dramatic declinations, frequently exceeding 90–99% by day 14 in amaranth, spinach, chili pepper, dried onion, and brewer’s yeast under light exposure, because the assay is highly sensitive to fast hydrogen-atom donors (catechols, thiols, ascorbic acid) that are rapidly consumed during early oxidative polymerization [53]. TEAC values declined more gradually, typically 25–57% after 14 days, indicating that many polymerized or partially oxidized phenolics preserve single-electron transfer ability even after losing rapid H-atom donation capacity [48]. FRAP values exhibited consistent, time-dependent reduction across virtually all samples (60–80% loss by day 14) with relatively modest influence of light, mirroring the progressive oxidation of phenolic hydroxyl groups responsible for Fe^3+^ reduction. The extreme DPPH instability of brewer’s yeast flakes and amaranth, despite a moderate decrease in the TPC, suggests the rapid destruction of non-phenolic reductants such as ergothioneine, reduced glutathione, or Maillard-derived compounds in the methanolic solution.

Light exposure at room temperature consistently accelerated the degradation of antioxidants to a larger extent than the implication of elevated (ambient) temperature alone, particularly for DPPH activity and TFC in curcuminoid- and quercetin-rich extracts. Photochemical mechanisms include direct excitation of conjugated systems leading to isomerization (trans- to cis-curcumin), cleavage, or formation of reactive oxygen species, as well as photosensitisation by residual chlorophyll or riboflavin, generating singlet oxygen that selectively attacks electron-rich phenolic rings [32,34]. Refrigeration at 4 °C markedly slowed down all the oxidative reactions, confirming the importance of low temperature even in solution to preserve biological activity [42], whereas dark ambient storage provided intermediate retaining antioxidants.

### 3.2. Possible Mechanisms for Degradation of Antioxidants

The most striking observation is the extremely rapid TPC decline in pigment-rich extracts: a 68–75% decrease is accomplished already after 3 days and >86% after 14 days, largely independent of storage temperature or light. Such fast degradation of phenolic compounds in methanol solutions has rarely been documented over such short periods. Several mechanisms may plausibly operate simultaneously: (1) Oxidative polymerization and precipitation: Even in pure methanol, residual oxygen dissolved in the solvent and headspace may be sufficient to initiate polyphenol oxidation. Phenolic compounds, especially of catechol- and pyrogallol-type structures, being abundant in basil, spinach, beetroot, and onion (chlorogenic acid, quercetin glycosides, protocatechuic acid), are likely prone to enzymatic and non-enzymatic oxidation, potentially yielding o-quinones, which may be rapidly polymerized into insoluble melanoid pigments [31,32]. Visual inspection of the stored extracts confirmed progressive browning and turbidity, particularly in basil, spinach, and onion samples—a classic sign of oxidative polymerization leading to loss of Folin-reactivity. (2) Co-pigmentation breakdown and metal-catalyzed oxidation: Many of the highly unstable extracts contain chlorophyll, betalains, or anthocyanin-derived structures that form transient co-pigmentation complexes with phenolics. Disruption of these complexes upon extraction releases free phenolics that are far more susceptible to oxidation [34]. Trace (transition) metals (Fe, Cu) leached from plant material can dramatically accelerate Fenton-type reactions in methanolic solution [36]. (3) Acid-catalyzed degradation in methanolic medium: Methanol itself can act as a weak acid catalyst, and residual plant organic acids lower the pH of the extracts (often 4.0–5.5 in our measurements). Under mildly acidic conditions combined with oxygen, many phenolic acids undergo decarboxylation or esterification reactions, reducing Folin–Ciocalteu reactivity [37].

In contrast, rosemary, thyme, oregano, and turmeric showed much slower decline in the TPC values (only 34–52% after 14 days). These species are dominated by highly stable phenolic diterpenes (carnosic acid, carnosol) and rosmarinic acid, which are known for intrinsic antioxidant protection and resistance to oxidation even in solution [46,48]. Carnosic acid, in particular, acts as a sacrificial antioxidant, regenerating itself via intramolecular cyclisation and protecting other phenolics [50].

Our results differ markedly from some earlier, short-term stability studies performed on solid plant materials or aqueous extracts. For example, ref. [44] observed an increase in caffeic acid and total phenolics in Lamiaceae infusions during 27 days of storage at room temperature, attributed to continued hydrolysis of complex esters. In contrast, we observed only a continuous decline in the methanol solution, highlighting that extraction into organic solvent dramatically accelerates degradation kinetics compared to the protective matrix of the dried plant.

While these hypotheses are consistent with the observed degradation patterns and the existing literature, targeted analytical approaches (e.g., LC–MS-based compound tracking or oxygen-controlled experiments) would be required to conclusively verify the specific pathways involved.

### 3.3. Practical Implications for Analytical Protocols

From a practical analytical perspective, the results demonstrate that methanol extracts of most of the plant materials, and especially pigment-rich vegetable-derived ones, cannot be stored longer than a few hours without substantial loss of the measured phenolic and antioxidant compounds. Even storage under refrigeration only delays but does not prevent significant degradation of the antioxidants over 7–14 days. Laboratories analyzing basil, spinach, beetroot, onion, tomato, or similar materials must perform assays immediately after extraction, ideally within 24 h, and should consider filling airtight vials or nitrogen flushing in case of any unavoidable delay. The extreme instability documented here for pigment-rich extracts in methanol has not been previously reported in such comprehensive short-term detail and represents a critical methodological consideration for future antioxidant screening studies. An additional methodological aspect that should be considered when interpreting the present results is the exclusive use of 100% methanol as the extraction solvent. Solvent composition strongly influences both the extraction efficiency and post-extraction stability of phenolic compounds by modifying polarity, oxygen solubility, co-extracted matrix components, and reaction kinetics. Consequently, the degradation patterns reported here may differ quantitatively from those occurring in aqueous or hydroalcoholic extracts frequently applied in food and plant biochemistry studies. The conclusions of the present work should therefore be interpreted primarily in relation to methanolic extracts. Future investigations will address solvent-dependent stability differences by comparing alternative solvent systems and solvent concentrations.

In comparison with earlier research focused on solid plant materials or aqueous extracts, it might be established that methanolic solutions are dramatically less stable. Dried rosemary or oregano may retain their antioxidant capacity for months or years, yet their methanol extracts lose their activity within days once the protective cellular matrix and natural synergists are removed. The rapid and extensive degradation of antioxidants observed in pigment-rich vegetable extracts therefore constitutes a novel and practically important finding that underscores the necessity of standardized immediate post-extraction analysis to ensure accurate and reproducible characterization of plants’ antioxidant potential across diverse matrices.

These results indicate that antioxidant-related metrics (TPC, TFC, DPPH, TEAC, FRAP) should not be viewed as fixed properties of plant materials, but rather as context-dependent outcomes influenced by the extract matrix and analytical approach. Variations in compound composition, co-extracted components, and their interactions can significantly affect both degradation behavior and assay responses. As a result, inconsistencies across studies may often reflect methodological differences—such as extraction procedures, storage conditions, and analytical timing—rather than true biological variability.

It should also be noted that the experimental time points begin at 3 days post-extraction. Although baseline measurements were performed immediately after extraction (Table 1), the study does not capture changes occurring within the first hours or within 24 h. Accordingly, claims regarding the necessity of “immediate” analysis should be interpreted with caution. Instead, the data demonstrate that substantial, matrix-dependent changes arise during short-term storage and are already evident by day 3 in many samples. This supports minimizing storage duration and standardizing analytical timing, while highlighting the need for higher-resolution studies to better characterize early-stage changes.

## 4. Materials and Methods

### 4.1. Sample Collection

A total of 15 distinctive spice and herb varieties, noted for elevated antioxidant potential, were procured from commercial outlets in Hungary. These included rosemary, thyme, oregano, basil, turmeric (powdered), beetroot (powdered), amaranth, spinach (powdered), dried onion, tomato (powdered), chili pepper, red pepper seasoning, yarrow tail, walnut (flour), and brewer’s yeast (flakes). Comprehensive details on common names, binomial nomenclature, taxonomic families, and utilized plant portions are provided in Table S1 (Supplementary Information).

### 4.2. Reagents and Standards

Folin–Ciocalteu phenol reagent was obtained from Chem-Lab NV (Zedelgem, Belgium). Gallic acid, sodium carbonate, methanol (≥99.8%), and rutin were sourced from Sigma-Aldrich (St. Louis, MO, USA). Sodium nitrite, aluminium chloride, sodium hydroxide, DPPH (2,2-diphenyl-1-picrylhydrazyl), Trolox (6-hydroxy-2,5,7,8-tetramethylchroman-2-carboxylic acid), ABTS (2,2′-azino-bis(3-ethylbenzothiazoline-6-sulfonic acid)), potassium persulfate, and TPTZ (2,4,6-tripyridyl-s-triazine) were acquired from Thermo Fisher Scientific (Shanghai, China). Acetone, acetate buffer, ascorbic acid, ferric chloride, and ferric chloride hexahydrate (FeCl_3_·6H_2_O) were also purchased from Sigma-Aldrich (St. Louis, MO, USA).

### 4.3. Quantification of Total Phenolic and Flavonoid Levels

Sample extraction involved mixing materials with pure methanol at a 1:10 (*w*/*v*) ratio. Mixtures underwent sonication for 30 min in an ultrasonic water bath, followed by centrifugation at 13,000× *g* for 3 min to isolate clear supernatants. Quadruplicate analyses were conducted for all assays.

The total phenolic content (TPC) of the extracts was assessed via the Folin–Ciocalteu colorimetric approach, adapted from Singleton and Rossi [54], with absorbance readings taken at 760 nm on a Shimadzu UV-160A spectrophotometer (Kyoto, Japan). Calibration relied on gallic acid standards, and results were calculated as mg gallic acid equivalents (GAE) per gram of dry weight (DW). The total flavonoid content (TFC) was evaluated by complexation with 10% aluminium chloride, measuring absorbance at 415 nm [55]. A rutin-based standard curve was employed, expressing outcomes as mg rutin per gram DW.

### 4.4. Evaluation of Antioxidant Potential

For antioxidant assays, pure methanol was used as the solvent at a sample-to-solvent ratio of 1:10 (*w*/*v*). Extracts were held at ambient temperature in darkness for 10 min prior to centrifugation at 13,000× *g* for 3 min to yield supernatants. All determinations were replicated four times.

#### 4.4.1. DPPH Radical-Scavenging Assay

Radical-scavenging activity was measured using well-established DPPH protocol [56]. A 0.23 mM DPPH solution was prepared by dissolving 9 mg in 100 mL methanol. One milliliter of the extract was combined with 2 mL of DPPH solution and incubated in darkness at room temperature for 30 min. Absorbance was recorded at 517 nm on the Shimadzu UV-160A spectrophotometer, with pure DPPH as the control. Trolox (10 mg in 10 mL methanol) served as the reference for calibration, and activity was reported as mg Trolox equivalents (TE) per gram DW.

#### 4.4.2. ABTS Radical Cation Decolorization (TEAC) Assay

Antioxidant power was determined through the long-established TEAC method [57], using extracts from Section 2.3. The ABTS•^+^ radical was generated by equimolar mixing of 7 mM ABTS and 5 mM potassium persulfate, followed by 12 h dark incubation. This stock was diluted 1:10 prior to use. The assay mixture consisted of 900 µL diluted ABTS•^+^, 1000 µL distilled water, and 100 µL extract (methanol substituted in blanks). After 20 min long dark incubation at room temperature, absorbance was measured at 734 nm. Trolox calibration was accomplished as presented for the DPPH assay, with results in mg TE per gram DW.

#### 4.4.3. Ferric Reducing Antioxidant Power (FRAP) Assay

The reducing capacity was quantified via the FRAP technique [58]. The working reagent was composed of 25 mL acetate buffer (300 mM, pH 3.6), 10 mL FeCl_3_·6H_2_O (20 mM), and 10 mL TPTZ (10 mM). Ten microliters of extract were added to 65 µL distilled water and 2250 µL of the reagent (distilled water for blanks), incubated for 8 min at room temperature. The absorbance was measured at 593 nm. Ascorbic acid (5.67 mM) provided the standard curve, yielding results as mg ascorbic acid equivalents per gram DW.

### 4.5. Storage Stability Trial

Post-extraction methanol supernatants were aliquot into Corning^®^ CentriStar™ Falcon tubes and subjected to three storage durations (3, 7, and 14 days) under varied conditions: refrigeration (4 °C in a Whirlpool W7X 820 OX unit, European Appliances Hungary Kft., Budapest, Hungary), dark ambient (22 ± 2 °C in a light-excluded cabinet), or light-exposed ambient (samples were stored at room temperature “22 ± 2 °C” under ambient laboratory daylight conditions in an indoor location receiving diffuse natural light but without direct sunlight exposure during the storage period.). The TPC, TFC, and antioxidant assays (DPPH, TEAC, FRAP) were performed on the stored samples to evaluate stability.

### 4.6. Statistical Analysis

Data were processed using IBM SPSS Statistics (version 28.0; IBM Corp., Armonk, NY, USA). Normality was verified via Shapiro–Wilk tests, and homogeneity of variances confirmed with Levene’s test. The effects of storage duration, condition, and their interaction on TPC, TFC, DPPH, TEAC, and FRAP values were analyzed by two-way ANOVA, with plant species as a blocking factor. Post hoc comparisons employed Tukey’s HSD test at *p* < 0.05. Percentage changes relative to fresh extracts (day 0) were calculated to quantify the extent of the degradation or the stability of the bioactives. Pearson correlation coefficients (r) assessed relationships between TPC/TFC and antioxidant metrics across fresh and stored samples. Principal component analysis (PCA) was applied to visualize multivariate patterns in antioxidant stability. Figures were generated in SPSS, including boxplots for storage effects and scatterplots for correlations. All analyses implied quadruplicate measurements, with results presented as mean ± standard deviation.

## 5. Conclusions

The present study demonstrates that methanolic extracts of antioxidant-rich plants exhibit markedly limited stability compared with dried herbs or aqueous infusions reported in the previous literature. Within 14 days, pigment-rich vegetable extracts (basil, beetroot, spinach, onion, tomato, and yarrow) showed severe degradation, with losses of 86–89% in total phenolic content (TPC) and up to 99% reduction in DPPH radical-scavenging activity, independent of storage conditions. In contrast, Lamiaceae herbs and turmeric retained antioxidant activity more effectively, with TPC reductions of 43.7% in rosemary, 50.6% in thyme, and 42.9% in oregano. Across all samples, refrigeration at 4 °C provided the greatest protection, whereas light-exposed room temperature conditions accelerated degradation most strongly.

These pronounced, species-dependent declines are likely associated with oxidative polymerization, disruption of co-pigment complexes, and light-induced reactions in solution. The findings indicate that methanolic extracts—particularly from leafy greens, bulbs, roots, and pigment-rich fruits—may undergo substantial compositional changes within hours to days, potentially leading to significant underestimation of bioactive potential if not analyzed promptly. Because 100% methanol was used exclusively as the extraction solvent, the observed stability patterns should be interpreted specifically within this solvent system. Variations in solvent polarity and composition may alter degradation kinetics, warranting further comparative studies using aqueous and hydroalcoholic systems.

For accurate quantification, spectrophotometric assays (TPC, TFC, DPPH, TEAC, FRAP) should ideally be performed within 24 h of extraction. When immediate analysis is not feasible, stabilization strategies such as storage at −20 °C or −80 °C, lyophilization, and the use of airtight, inert gas-flushed containers are recommended to minimize oxidative losses. Future research should incorporate alternative solvents, oxygen-limited conditions, ultra-low-temperature storage, and metabolomic approaches to identify labile compounds and improve preservation protocols.

Overall, antioxidant capacity and polyphenol content are strongly influenced by extraction matrix and methodological conditions rather than representing fixed plant properties. The results emphasize the necessity of standardized protocols and strict control of storage conditions to ensure reproducibility and reliable comparison across studies. Further high-resolution temporal studies are needed to capture early-stage post-extraction changes more precisely.

## Figures and Tables

**Table 1:** Total polyphenol content (TPC), total flavonoid content (TFC), and antioxidant capacity (DPPH, TEAC, and FRAP) in methanol extracts of 15 plant species—rosemary, thyme, oregano, basil, turmeric powder, beetroot powder, amaranth, spinach powder, dried onion, tomato powder, chili pepper, red pepper spice, yarrow tail, walnut flour, and brewer’s yeast flakes.

	TPC	TFC	DPPH	TEAC	FRAP
Rosemary	50.5 ± 0.44	23.7 ± 0.36	487 ± 1.81	10.2 ± 0.43	84.5 ± 3.15
Thyme	41.9 ± 0.36	27.6 ± 0.43	449 ± 1.47	9.7 ± 0.06	65.9 ± 0.84
Oregano	32.6 ± 1.96	18.1 ± 0.25	450 ± 0.50	8.8 ± 0.51	65.9 ± 1.35
Basil	24.2 ± 0.25	15.3 ± 0.32	340 ± 1.48	5.6 ± 0.21	65.1 ± 2.46
Turmeric powder	19.3 ± 0.55	148.9 ± 0.87	447 ± 1.43	9.9 ± 0.35	56.8 ± 1.62
Beetroot powder	7.8 ± 0.38	4.6 ± 0.06	294 ± 1.32	5.2 ± 0.14	40.9 ± 1.07
Amaranth	0.02 ± 0.006	0.4 ± 0.02	252 ± 2.25	1.2 ± 0.03	11.5 ± 0.20
Spinach powder	7.6 ± 0.60	6.1 ± 0.35	55 ± 5.15	4.4 ± 0.21	25.8 ± 0.99
Dried onion	4.5 ± 0.44	0.9 ± 0.04	41 ± 2.06	2.9 ± 0.05	11.0 ± 0.51
Tomato powder	16.9 ± 0.86	7.8 ± 0.26	112 ± 7.67	2.9 ± 0.15	18.9 ± 0.76
Chili pepper	8.3 ± 0.23	6.0 ± 0.59	265 ± 3.01	2.1 ± 0.07	15.1 ± 0.52
Red pepper spice	6.2 ± 0.61	22.5 ± 0.27	270 ± 4.21	4.9 ± 0.13	21.4 ± 0.73
Yarrow tail	25.1 ± 0.10	18.6 ± 0.11	438 ± 5.20	7.7 ± 0.10	49.4 ± 1.51
Walnut flour	29.9 ± 0.58	5.6 ± 0.39	424 ± 6.16	9.9 ± 0.42	55.6 ± 2.38
Brewer’s yeast flakes	0.8 ± 0.06	0.4 ± 0.04	24 ± 4.77	1.2 ± 0.10	11.9 ± 0.46

**Table 2:** Results of covariance analysis of the effect of storage period (3, 7, or 14 days) and storage conditions (refrigeration (4 °C), dark (22 ± 2 °C), or light-exposed ambient (shelf; 22 ± 2 °C)) on total polyphenol content (TPC), total flavonoid content (TFC), antioxidant capacity (DPPH, TEAC, and FRAP) in methanol extracts of 15 plant species—rosemary, thyme, oregano, basil, turmeric powder, beetroot powder, amaranth, spinach powder, dried onion, tomato powder, chili pepper, red pepper spice, yarrow tail, walnut flour, and brewer’s yeast flakes.

	TPC	TFC	DPPH	TEAC	FRAP
Species	3974.32 ***	848.13 ***	4843.82 ***	847.81 ***	157.05 ***
Days	6434.54 ***	3144.83 ***	3270.94 ***	1456.36 ***	8069.84 ***
Conditions	35.59 ***	51.40 ***	2426.74 ***	439.97 ***	142.42 ***
Days × Conditions	9.91 ***	40.01 ***	117.11 ***	5.93 ***	2.54 ***
Species × Days × Conditions	41.86 ***	27.45 ***	60.96 ***	16.83 ***	13.63 ***

*** significant at *p* ≥ 0.001 according to Tukey’s test.

## Data Availability

The original contributions presented in this study are included in the article material. Further inquiries can be directed to the corresponding author.

## Supplementary Materials

The following supporting information can be downloaded at: https://www.mdpi.com/article/10.3390/ijms27093723/s1.
