# Supplementary material for: Rapid, Matrix-Dependent Changes in Polyphenols and Antioxidant Capacity of Methanol Plant Extracts During Short-Term Storage: Implications for Analytical Timing
Source: Int J Mol Sci. 2026 Apr 22;27(9):3723. doi: 10.3390/ijms27093723 (PMC13164456; doi:10.3390/ijms27093723)
Supplement: Supplementary file 1 [file ijms-27-03723-s001.zip › ijms-4264017-supplementary.pdf]

Table S1: The plant names, scientific names, plant families, and used parts of the raw materials

	**Scientific name**	**Plant Family**	**Used part of raw material**
Rosemary	*Salvia rosmarinus* L.	Lamiaceae	leaves
Thyme	*Thymus vulgaris* L.	Lamiaceae	leaves
Oregano	*Origanum vulgare* L.	Lamiaceae	leaves
Basil	*Ocimum basilicum* L.	Lamiaceae	leaves
Turmeric powder	*Curcuma longa* L.	Zingiberaceae	rhizomes
Beetroot powder	*Beta vulgaris* L.	Amaranthaceae	taproot
Amaranth	*Amaranthus spp.*	Amaranthaceae	seeds
Spinach powder	*Spinacia oleracea* L.	Amaranthaceae	leaves
Dried onion	*Allium cepa* L.	Amaryllidaceae	bulbs
Tomato powder	*Solanum lycopersicum L.*	Solanaceae	fruits
Chili pepper	*Capsicum annuum* L.	Solanaceae	placenta
Red pepper	*Capsicum annuum* L.	Solanaceae	fruits
Yarrow tail	*Achillea millefolium* L.	Asteraceae	leaves
Walnut flour	*Juglans regia L.*	Juglandaceae	kernel
Brewer's yeast flakes	*Saccharomyces cerevisiae*	Saccharo-mycetaceae	one cell fungus

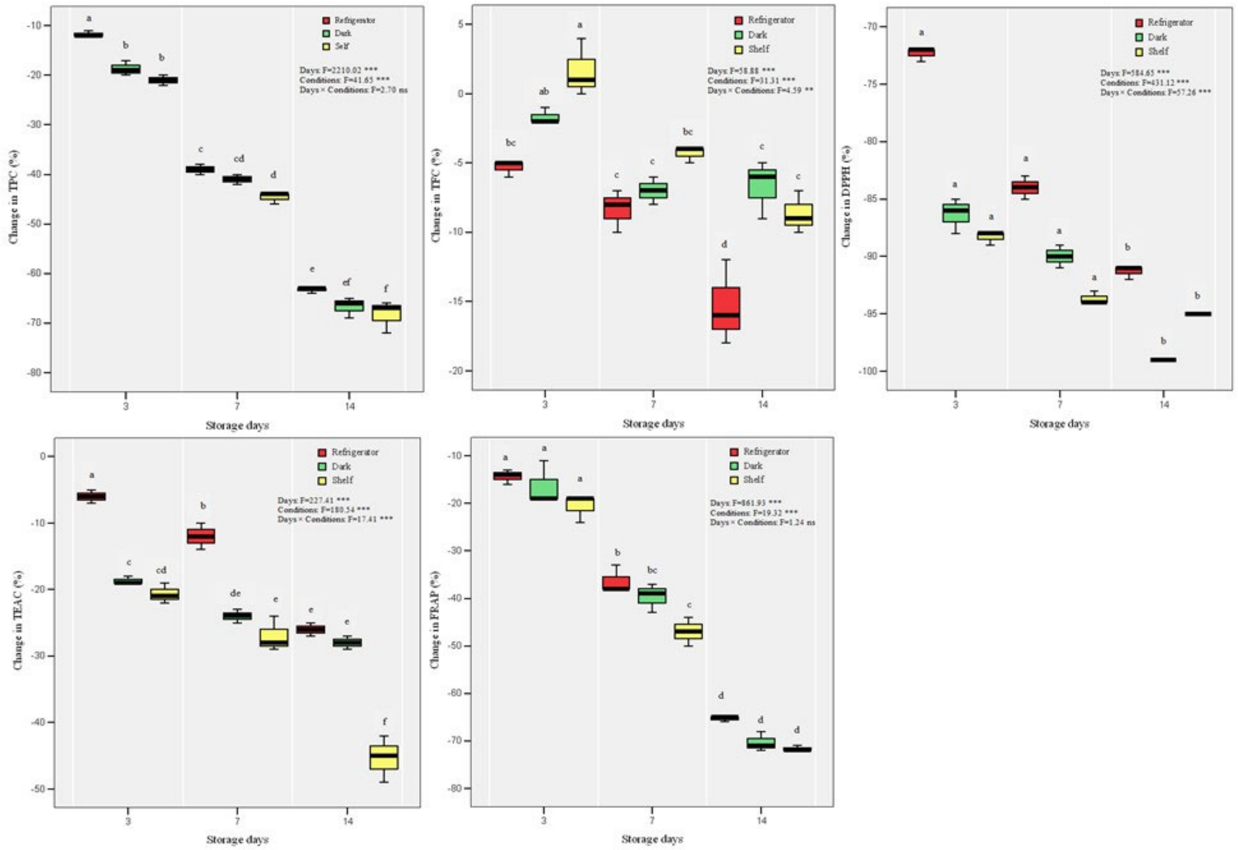

Figure S1: Changes in total polyphenol content, total flavonoid content (TFC), DPPH, TEAC, and FRAP in methanol extracts of rosemary after storage for 3, 7, or 14 days under varied conditions: refrigeration (4 °C in a Whirlpool W7X 820 OX unit), dark ambient (22 ± 2 °C in a light-excluded cabinet), or light-exposed ambient (shelf; 22 ± 2 °C near a window) for different periods. Values are means ± SD (n = 4).

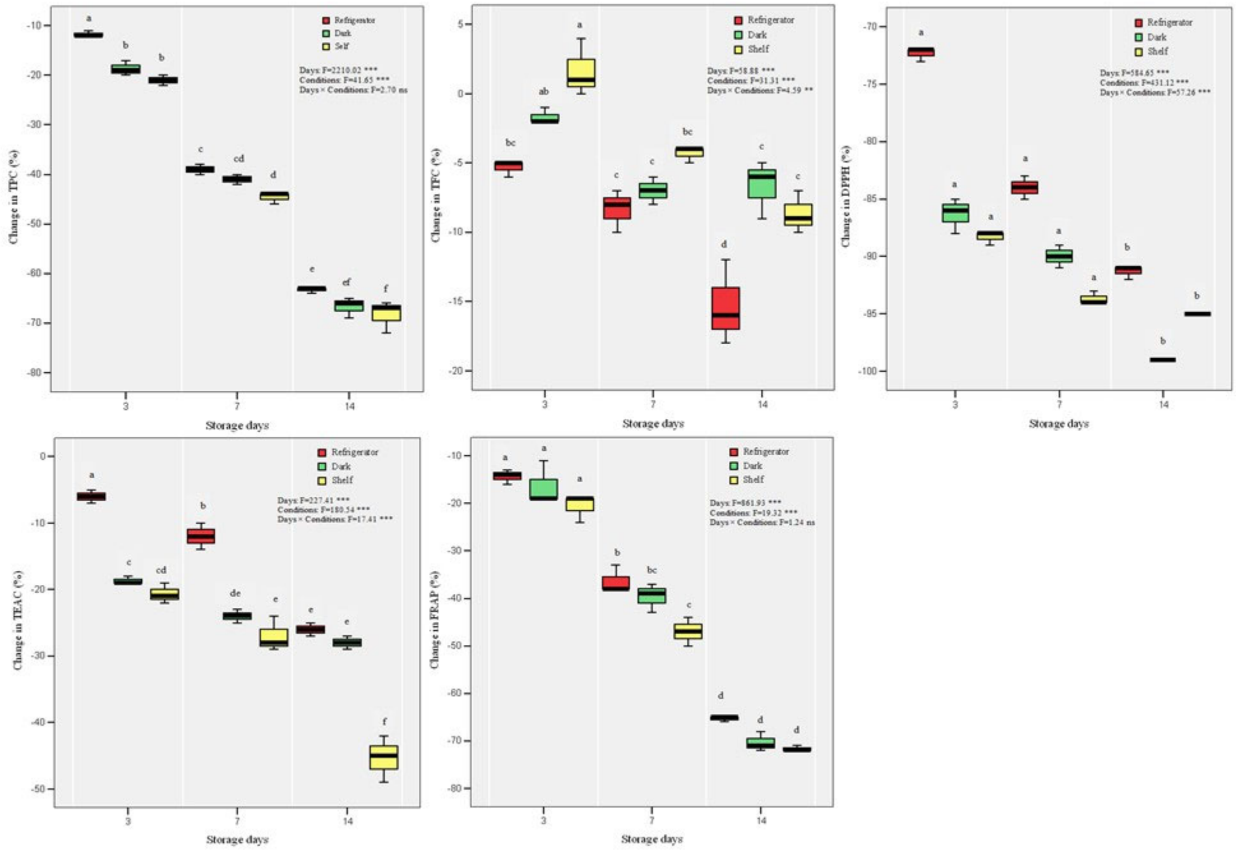

Figure S2: Changes in total polyphenol content, total flavonoid content (TFC), DPPH, TEAC, and FRAP in methanol extracts of amaranth after storage for 3, 7, or 14 days under varied conditions: refrigeration (4 °C in a Whirlpool W7X 820 OX unit), dark ambient (22 ± 2 °C in a light-excluded cabinet), or light-exposed ambient (shelf; 22 ± 2 °C near a window) for different periods. Values are means ± SD (n = 4).

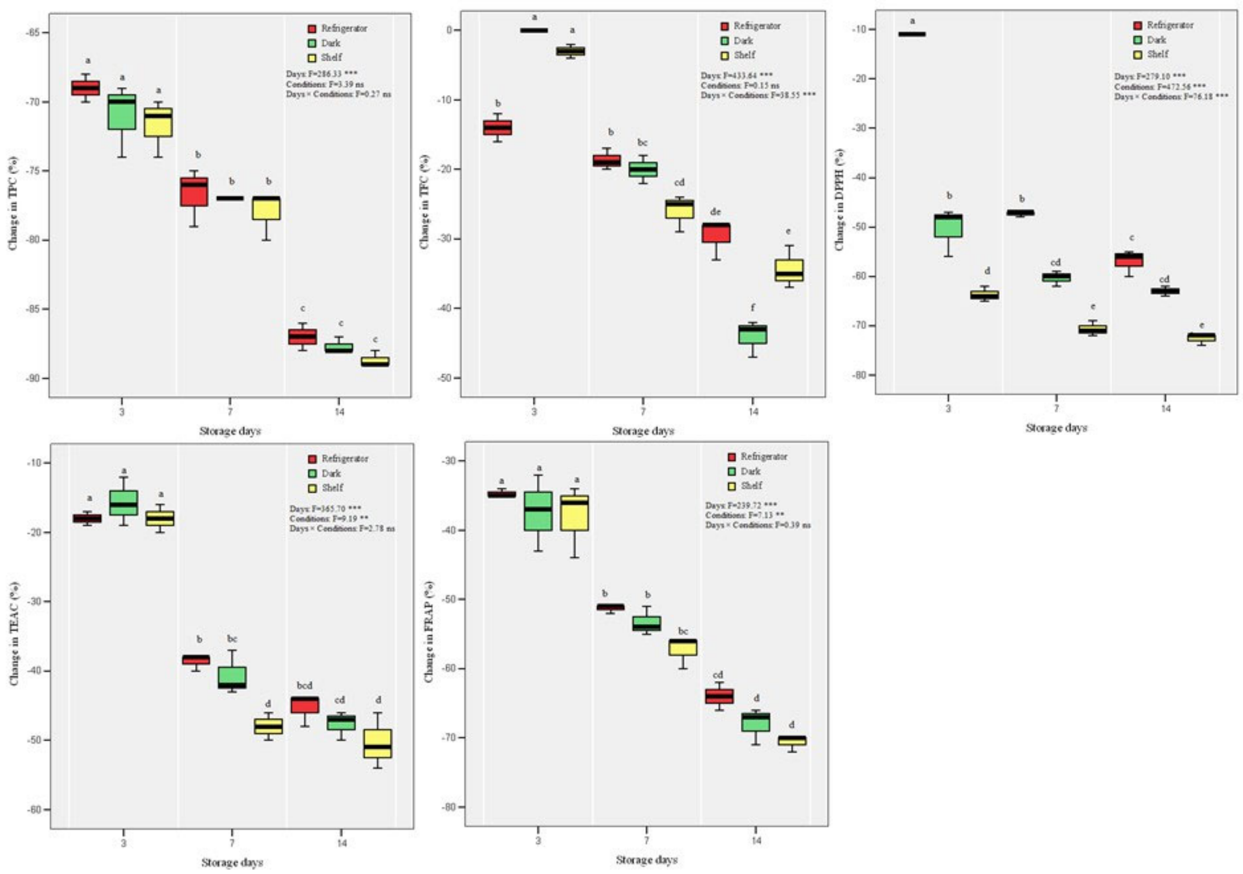

Figure S3: Changes in total polyphenol content, total flavonoid content (TFC), DPPH, TEAC, and FRAP in methanol extracts of basil after storage for 3, 7, or 14 days under varied conditions: refrigeration (4 °C in a Whirlpool W7X 820 OX unit), dark ambient (22 ± 2 °C in a light-excluded cabinet), or light-exposed ambient (shelf; 22 ± 2 °C near a window) for different periods. Values are means ± SD (n = 4).

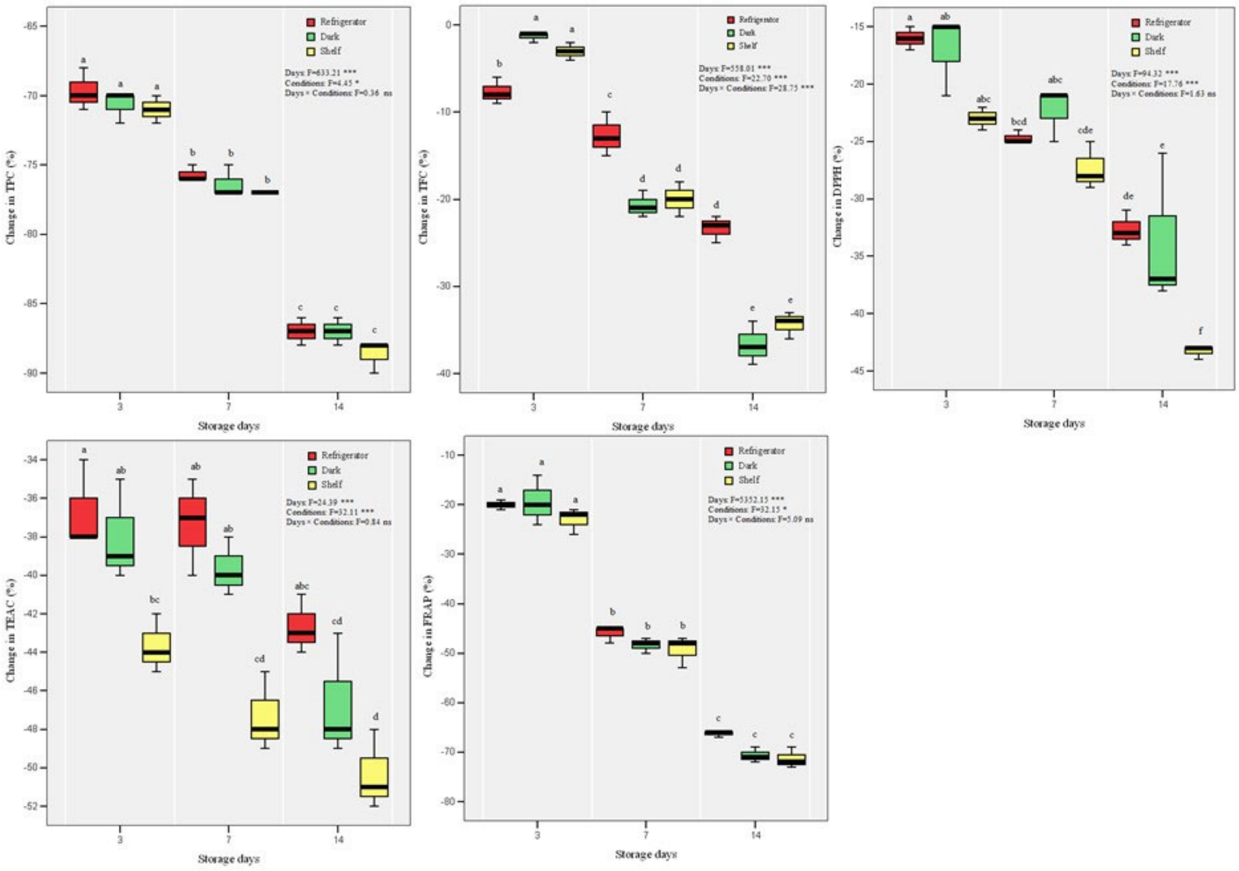

Figure S4: Changes in total polyphenol content, total flavonoid content (TFC), DPPH, TEAC, and FRAP in methanol extracts of beet root after storage for 3, 7, or 14 days under varied conditions: refrigeration (4 °C in a Whirlpool W7X 820 OX unit), dark ambient (22 ± 2 °C in a light-excluded cabinet), or light-exposed ambient (shelf; 22 ± 2 °C near a window) for different periods. Values are means ± SD (n = 4).

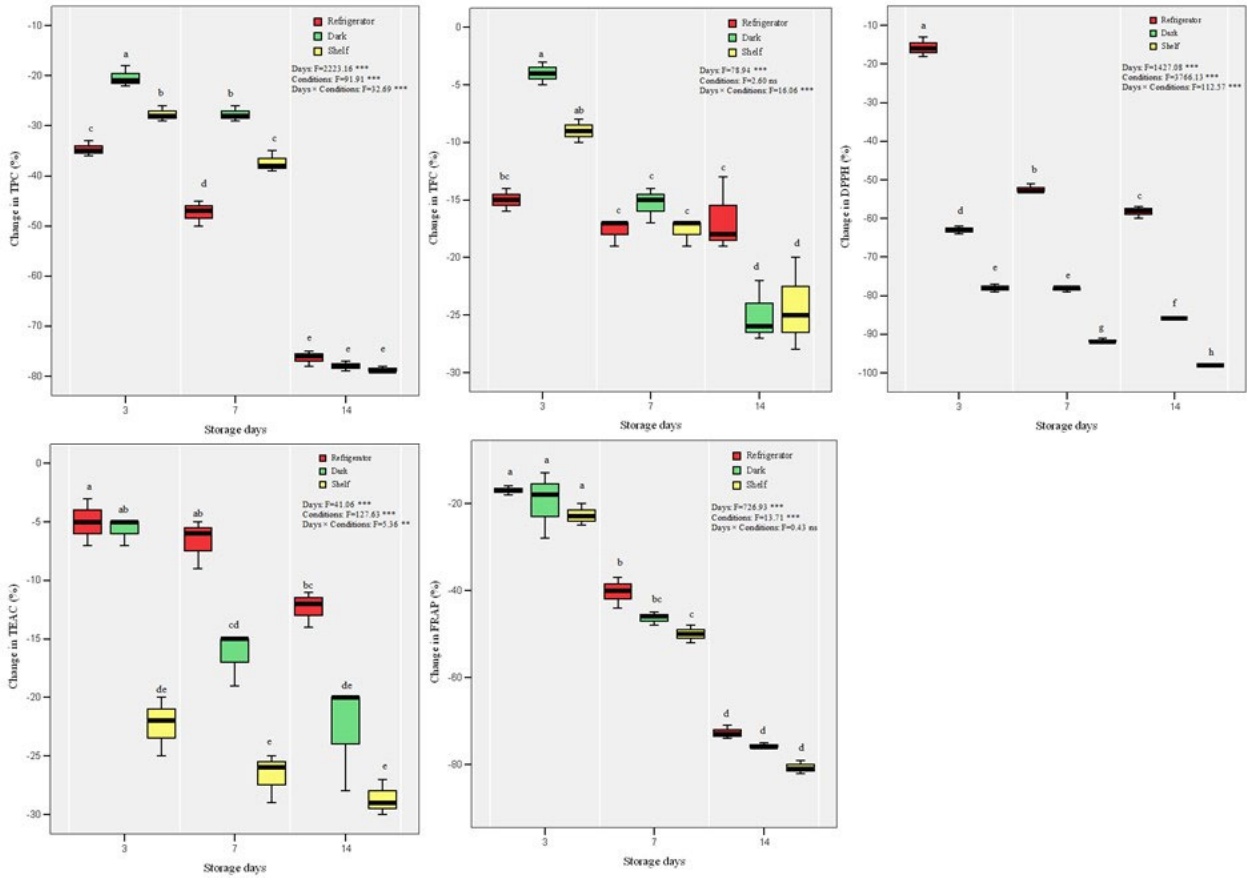

Figure S5: Changes in total polyphenol content, total flavonoid content (TFC), DPPH, TEAC, and FRAP in methanol extracts of chili pepper after storage for 3, 7, or 14 days under varied conditions: refrigeration (4 °C in a Whirlpool W7X 820 OX unit), dark ambient (22 ± 2 °C in a light-excluded cabinet), or light-exposed ambient (shelf; 22 ± 2 °C near a window) for different periods. Values are means ± SD (n = 4).

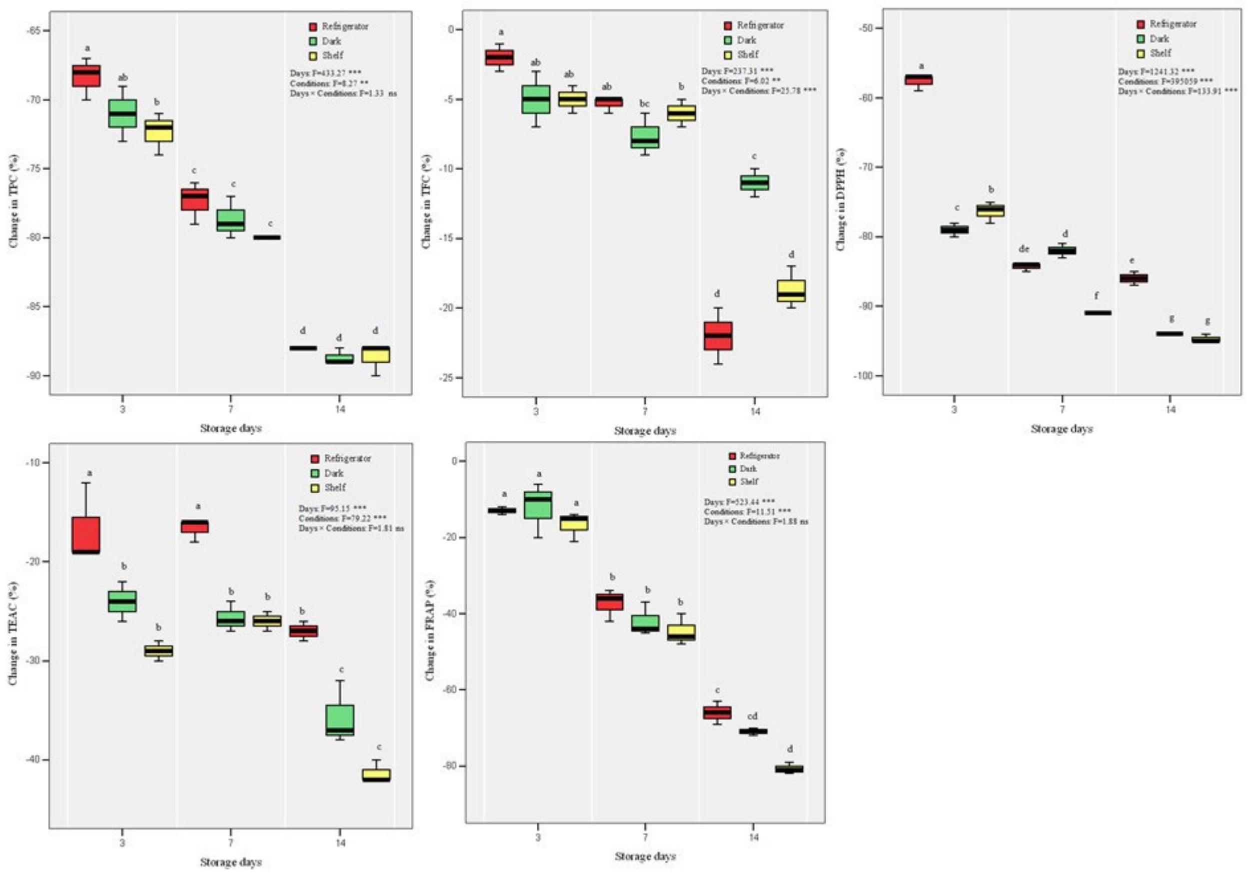

Figure S6: Changes in total polyphenol content, total flavonoid content (TFC), DPPH, TEAC, and FRAP in methanol extracts of dried onion after storage for 3, 7, or 14 days under varied conditions: refrigeration (4 °C in a Whirlpool W7X 820 OX unit), dark ambient (22 ± 2 °C in a light-excluded cabinet), or light-exposed ambient (shelf; 22 ± 2 °C near a window) for different periods. Values are means ± SD (n = 4).

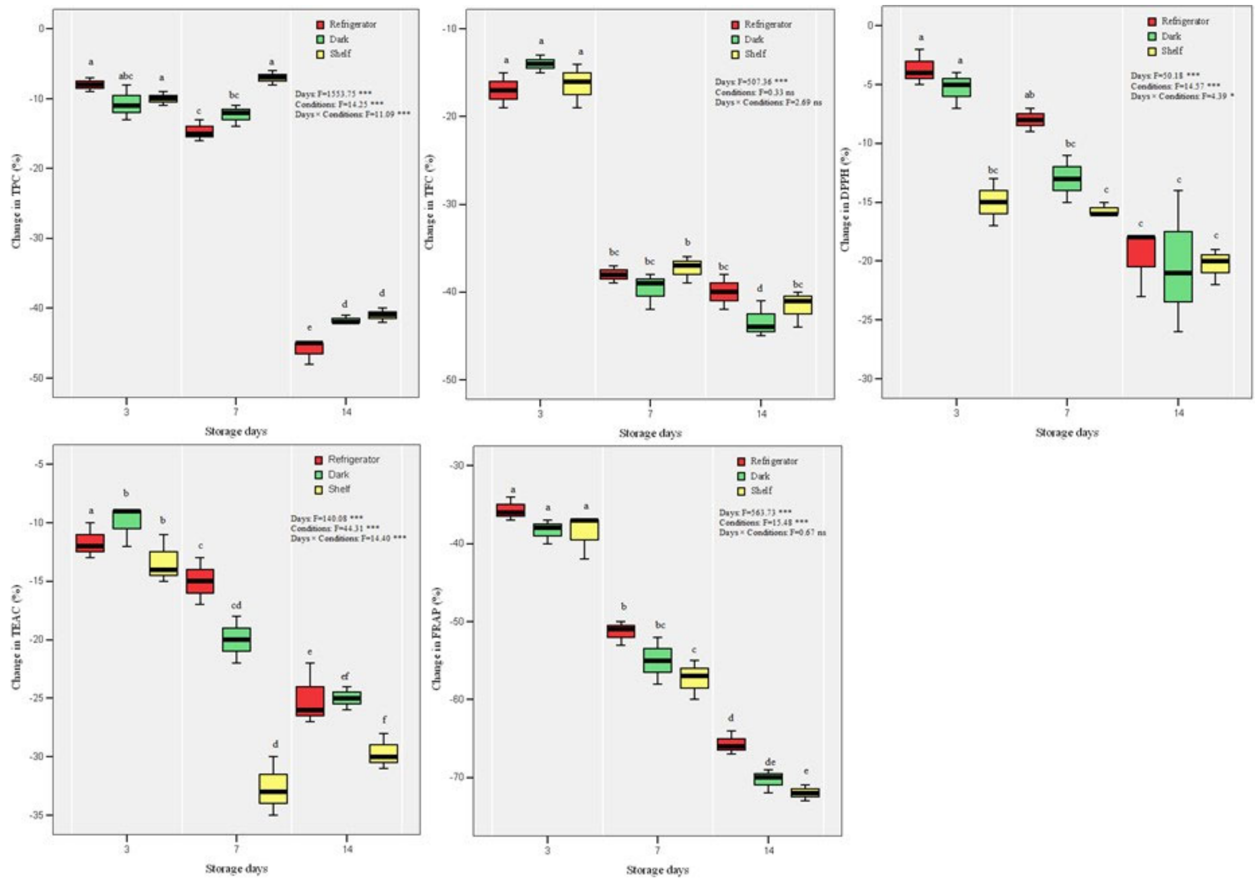

Figure S7: Changes in total polyphenol content, total flavonoid content (TFC), DPPH, TEAC, and FRAP in methanol extracts of oregano after storage for 3, 7, or 14 days under varied conditions: refrigeration (4 °C in a Whirlpool W7X 820 OX unit), dark ambient (22 ± 2 °C in a light-excluded cabinet), or light-exposed ambient (shelf; 22 ± 2 °C near a window) for different periods. Values are means ± SD (n = 4).

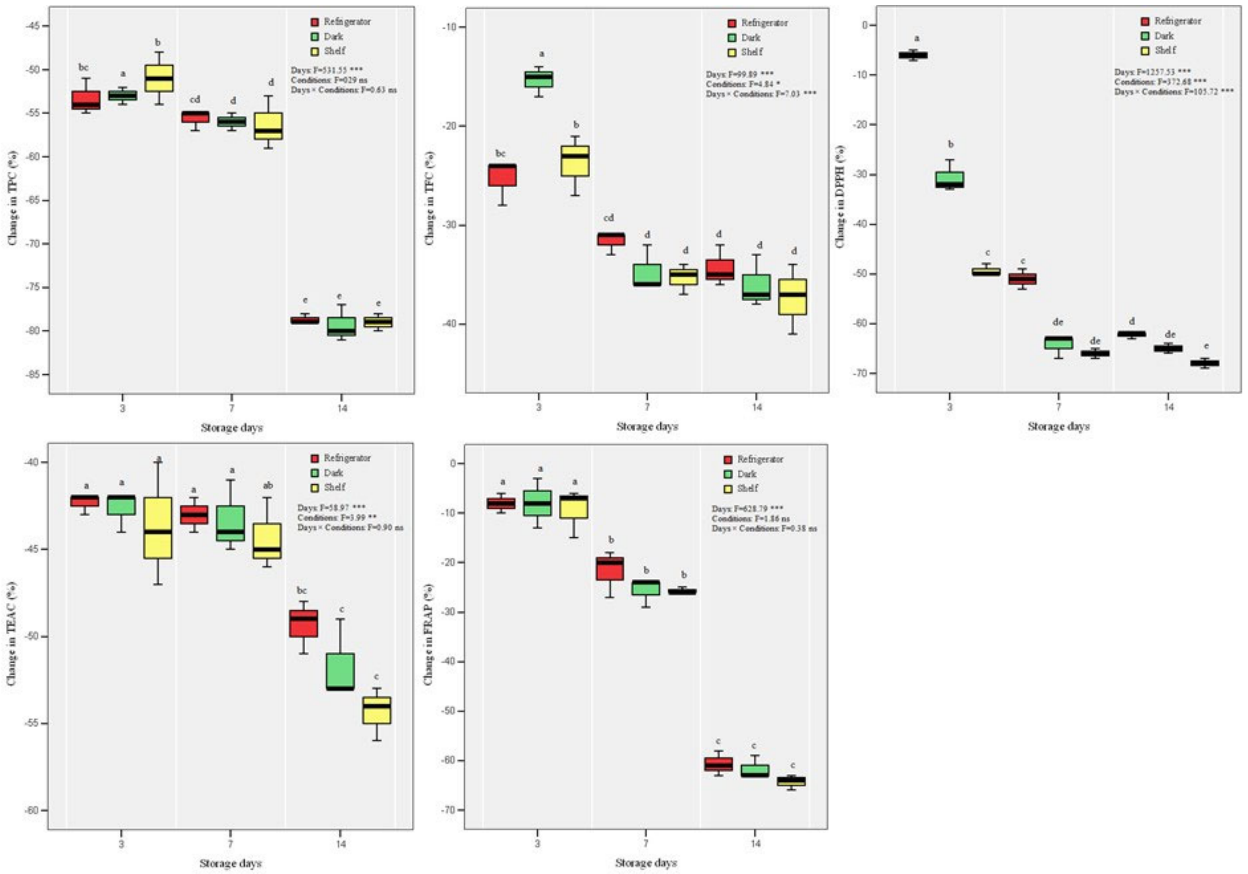

Figure S8: Changes in total polyphenol content, total flavonoid content (TFC), DPPH, TEAC, and FRAP in methanol extracts of red pepper after storage for 3, 7, or 14 days under varied conditions: refrigeration (4 °C in a Whirlpool W7X 820 OX unit), dark ambient (22 ± 2 °C in a light-excluded cabinet), or light-exposed ambient (shelf; 22 ± 2 °C near a window) for different periods. Values are means ± SD (n = 4).

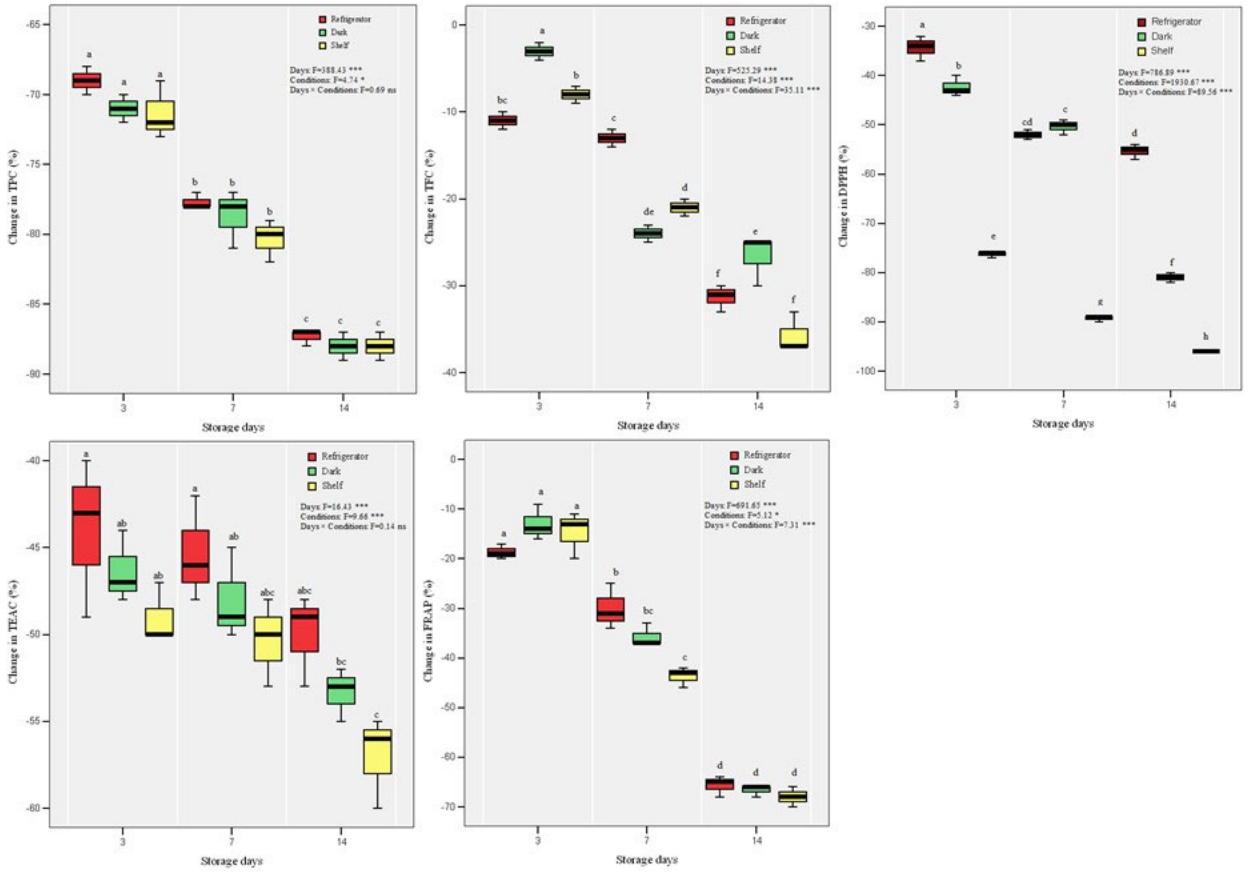

Figure S9: Changes in total polyphenol content, total flavonoid content (TFC), DPPH, TEAC, and FRAP in methanol extracts of spinach after storage for 3, 7, or 14 days under varied conditions: refrigeration (4 °C in a Whirlpool W7X 820 OX unit), dark ambient (22 ± 2 °C in a light-excluded cabinet), or light-exposed ambient (shelf; 22 ± 2 °C near a window) for different periods. Values are means ± SD (n = 4).

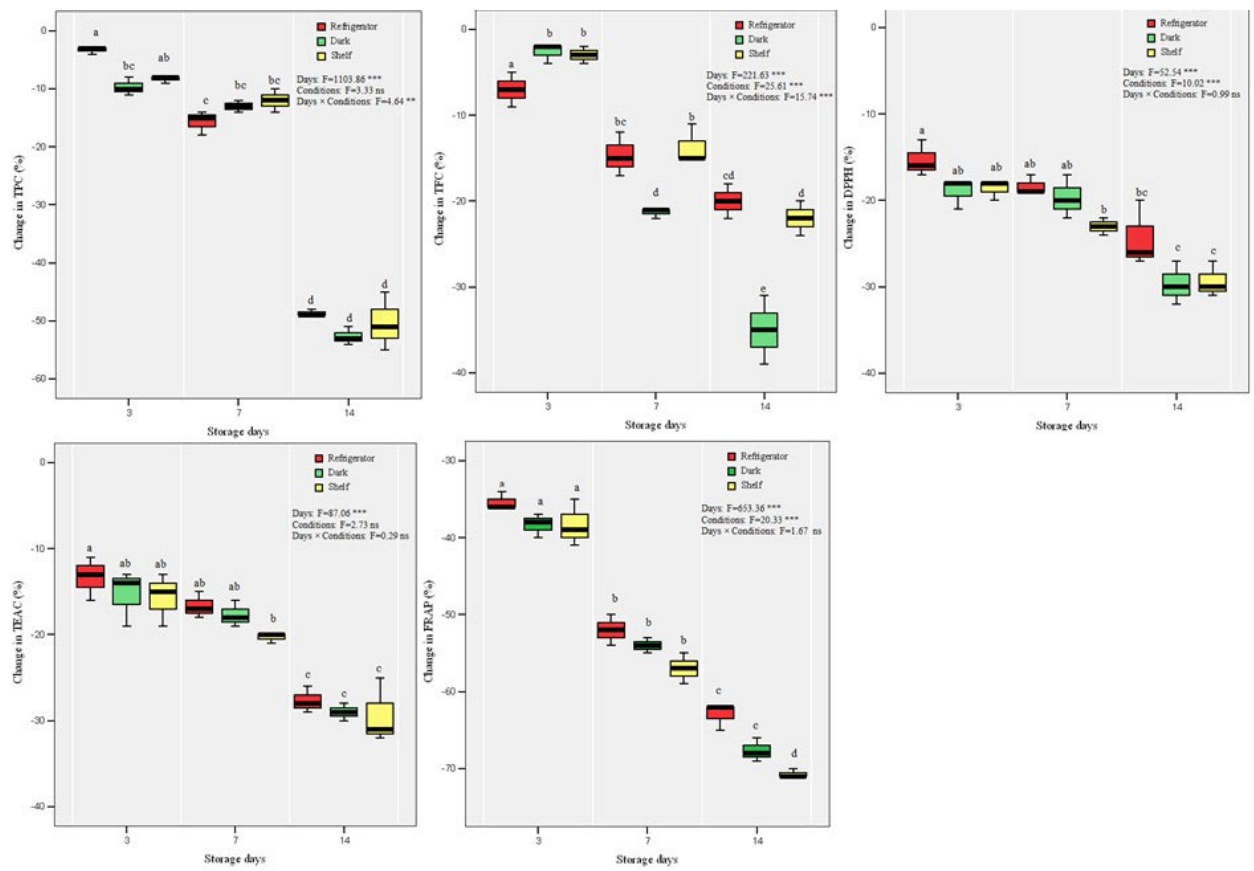

Figure S10: Changes in total polyphenol content, total flavonoid content (TFC), DPPH, TEAC, and FRAP in methanol extracts of thyme after storage for 3, 7, or 14 days under varied conditions: refrigeration (4 °C in a Whirlpool W7X 820 OX unit), dark ambient (22 ± 2 °C in a light-excluded cabinet), or light-exposed ambient (shelf; 22 ± 2 °C near a window) for different periods. Values are means ± SD (n = 4).

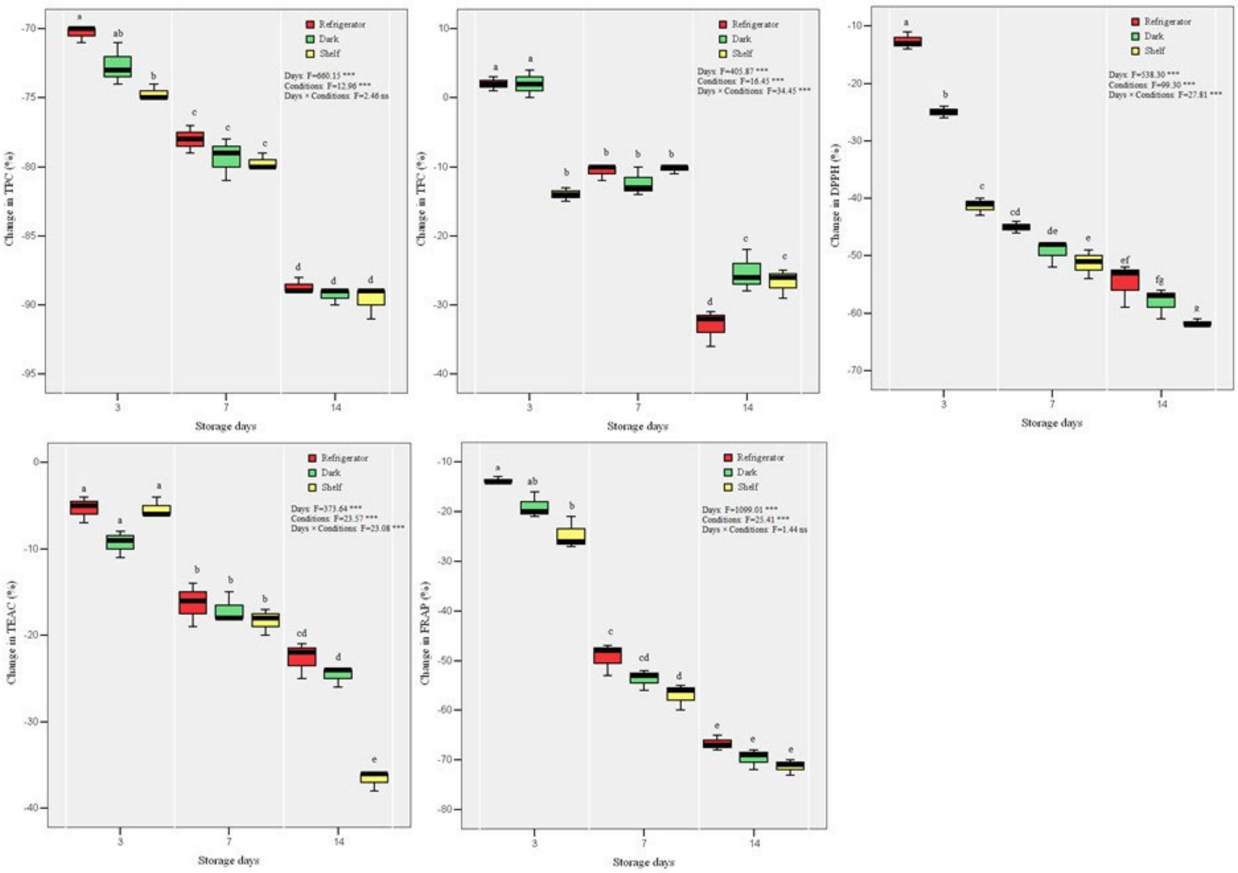

Figure S11: Changes in total polyphenol content, total flavonoid content (TFC), DPPH, TEAC, and FRAP in methanol extracts of tomato after storage for 3, 7, or 14 days under varied conditions: refrigeration (4 °C in a Whirlpool W7X 820 OX unit), dark ambient (22 ± 2 °C in a light-excluded cabinet), or light-exposed ambient (shelf; 22 ± 2 °C near a window) for different periods. Values are means ± SD (n = 4).

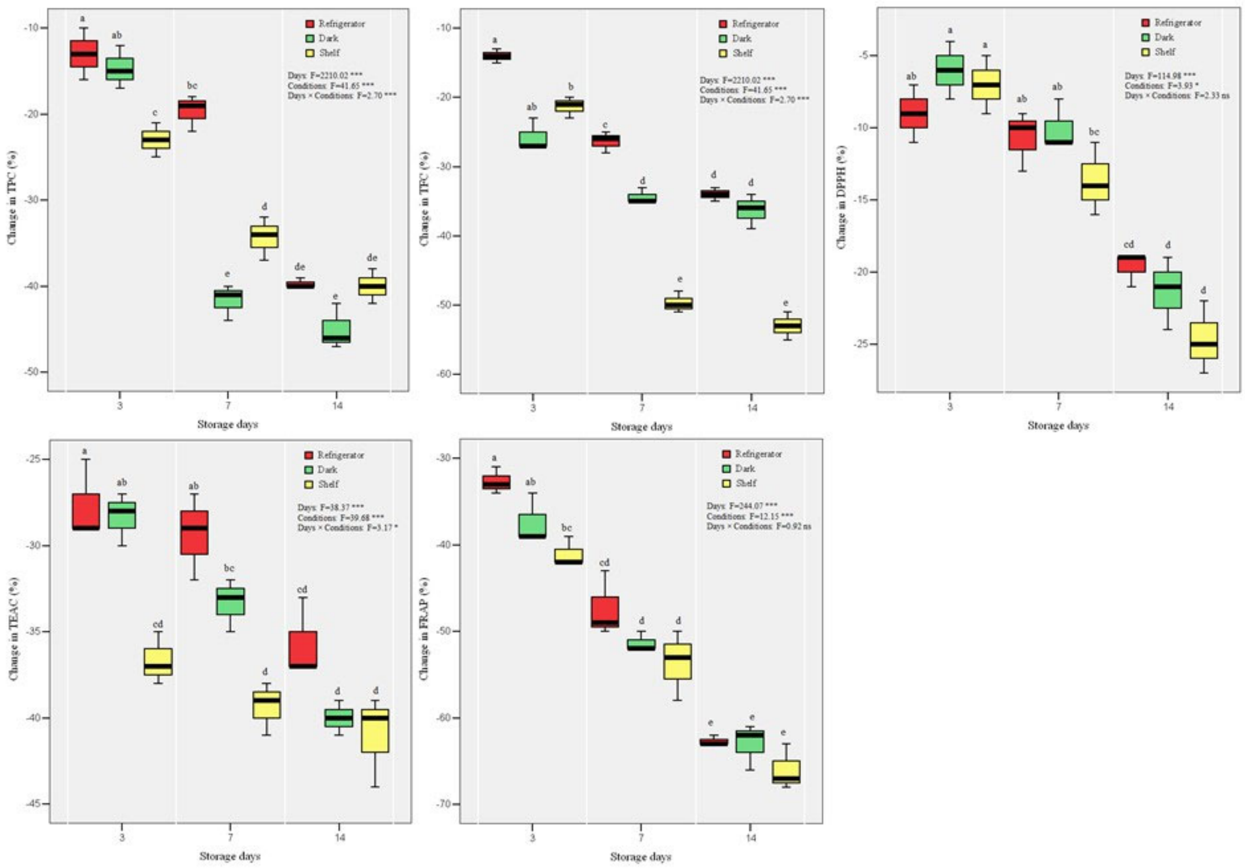

Figure S12: Changes in total polyphenol content, total flavonoid content (TFC), DPPH, TEAC, and FRAP in methanol extracts of tumeric powder after storage for 3, 7, or 14 days under varied conditions: refrigeration (4 °C in a Whirlpool W7X 820 OX unit), dark ambient (22 ± 2 °C in a light-excluded cabinet), or light-exposed ambient (shelf; 22 ± 2 °C near a window) for different periods. Values are means ± SD (n = 4).

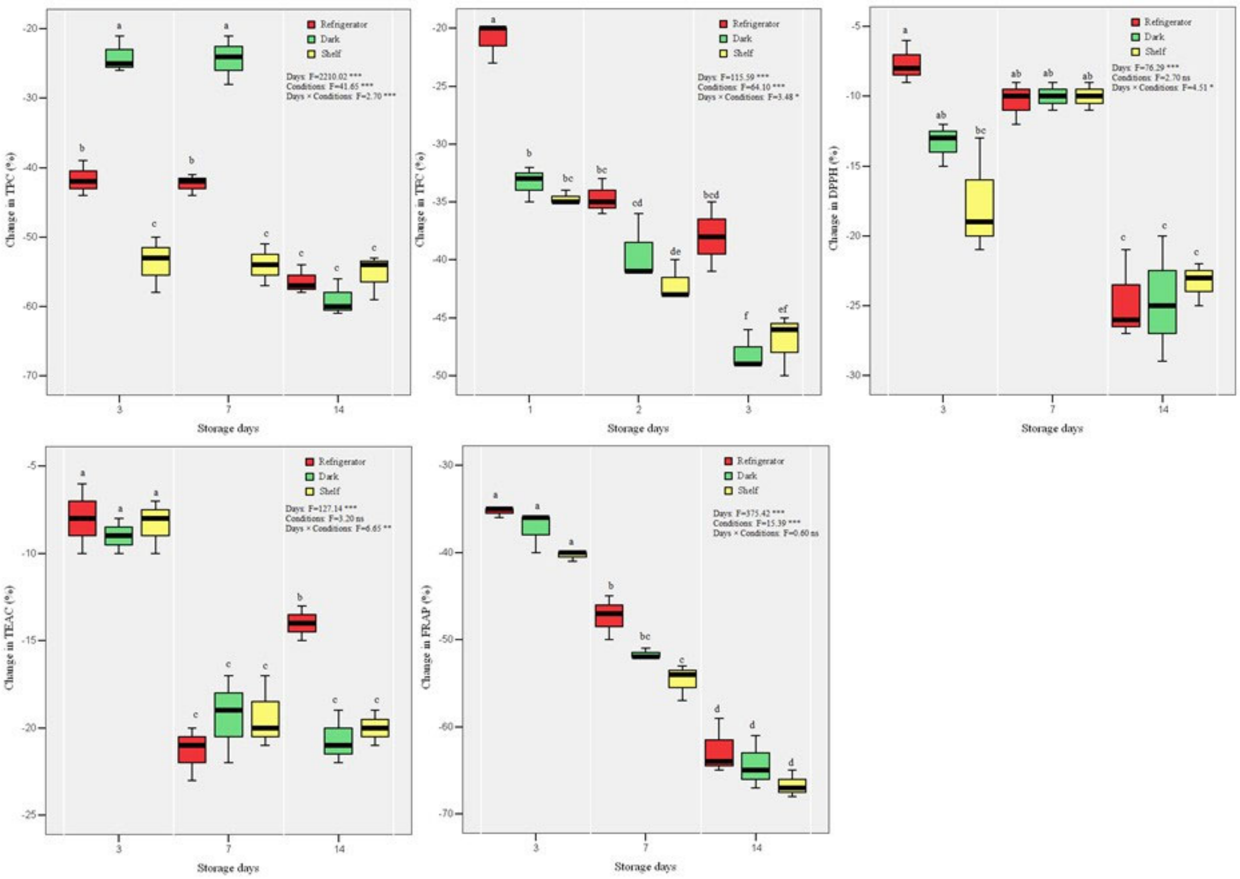

Figure S13: Changes in total polyphenol content, total flavonoid content (TFC), DPPH, TEAC, and FRAP in methanol extracts of walnut flour after storage for 3, 7, or 14 days under varied conditions: refrigeration (4 °C in a Whirlpool W7X 820 OX unit), dark ambient (22 ± 2 °C in a light-excluded cabinet), or light-exposed ambient (shelf; 22 ± 2 °C near a window) for different periods. Values are means ± SD (n = 4).

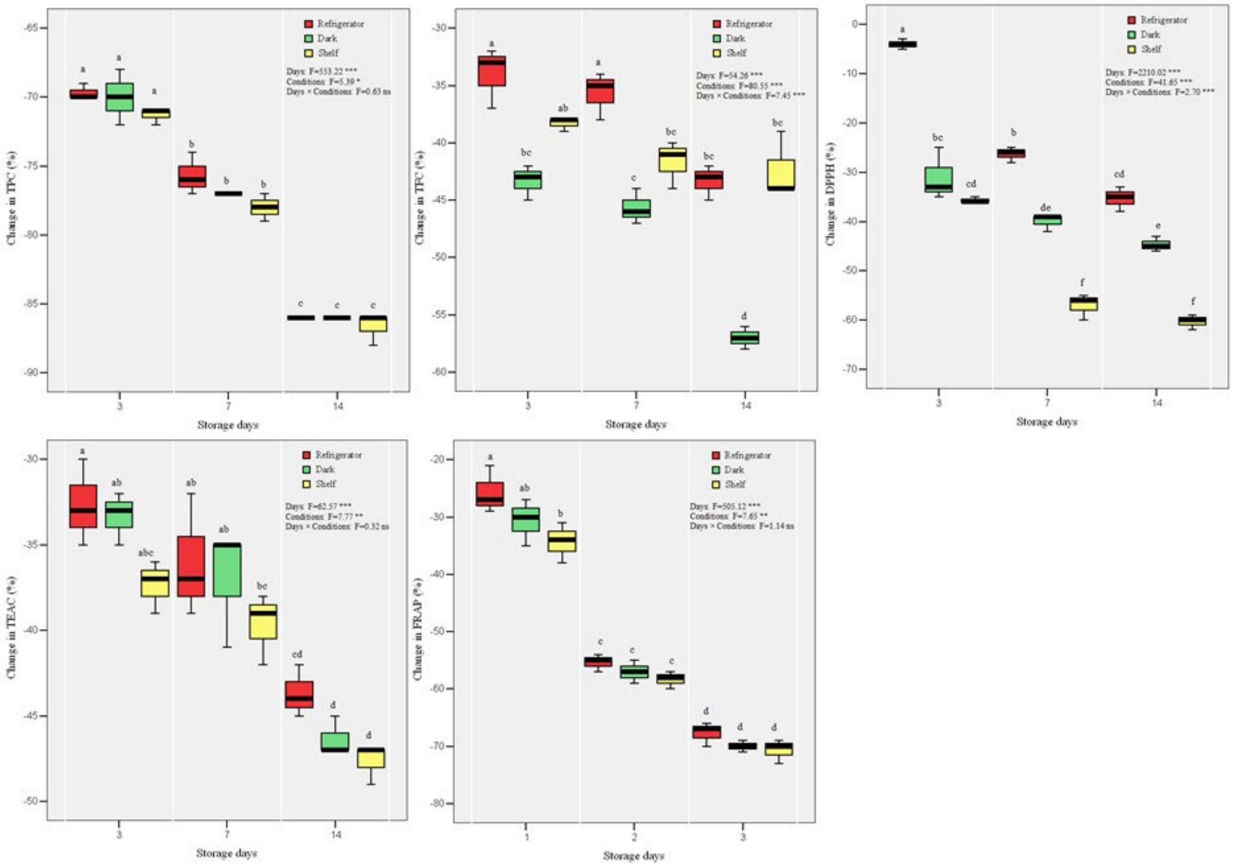

Figure S14: Changes in total polyphenol content, total flavonoid content (TFC), DPPH, TEAC, and FRAP in methanol extracts of yarrow tail after storage for 3, 7, or 14 days under varied conditions: refrigeration (4 °C in a Whirlpool W7X 820 OX unit), dark ambient (22 ± 2 °C in a light-excluded cabinet), or light-exposed ambient (shelf; 22 ± 2 °C near a window) for different periods. Values are means ± SD (n = 4).

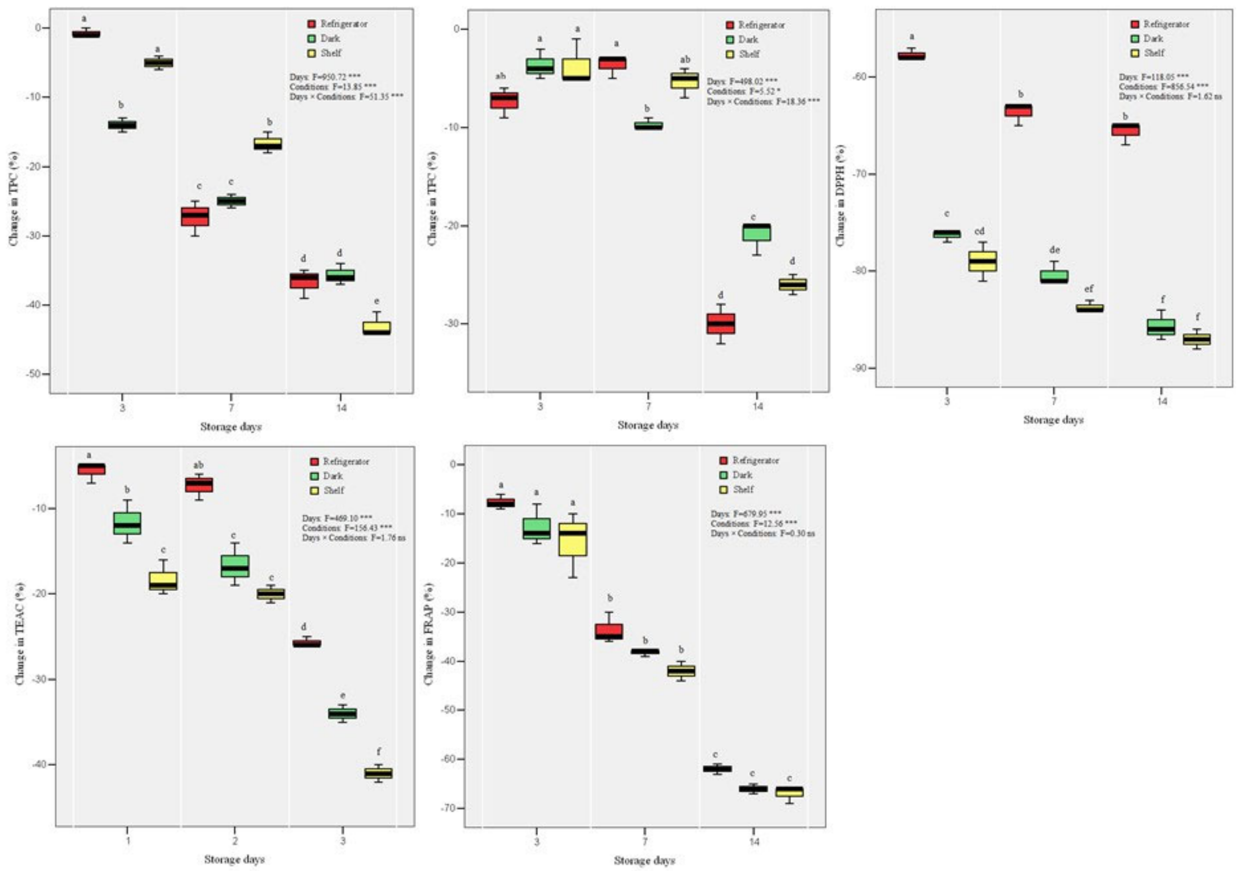

Figure S15: Changes in total polyphenol content, total flavonoid content (TFC), DPPH, TEAC, and FRAP in methanol extracts of brewer's yeast flakes after storage for 3, 7, or 14 days under varied conditions: refrigeration (4 °C in a Whirlpool W7X 820 OX unit), dark ambient (22 ± 2 °C in a light-excluded cabinet), or light-exposed ambient (shelf; 22 ± 2 °C near a window) for different periods. Values are means ± SD (n = 4).
